# Revisiting Platelets and Toll-Like Receptors (TLRs): At the Interface of Vascular Immunity and Thrombosis

**DOI:** 10.3390/ijms21176150

**Published:** 2020-08-26

**Authors:** Kathryn Hally, Sebastien Fauteux-Daniel, Hind Hamzeh-Cognasse, Peter Larsen, Fabrice Cognasse

**Affiliations:** 1 Department of Surgery and Anaesthesia, The University of Otago, Wellington 6021, New Zealand; peter.larsen@otago.ac.nz; 2 Wellington Cardiovascular Research Group, Wellington 6021, New Zealand; 3 School of Biological Sciences, Victoria University of Wellington, Wellington 6012, New Zealand; 4 Établissement Français du Sang Auvergne-Rhône-Alpes, 69150 Saint-Étienne, France; sebastien.fauteux@efs.sante.fr (S.F.-D.); fabrice.cognasse@efs.sante.fr (F.C.); 5 Faculté de Medecine Jacques Lisfranc, Université de Lyon, GIMAP-EA3064, 42270 Saint-Etienne, France; hamzeh@univ-st-etienne.fr

**Keywords:** platelets, Toll-like receptors (TLRs), thrombosis, inflammation, infection

## Abstract

While platelet function has traditionally been described in the context of maintaining vascular integrity, recent evidence suggests that platelets can modulate inflammation in a much more sophisticated and nuanced manner than previously thought. Some aspects of this expanded repertoire of platelet function are mediated via expression of Toll-like receptors (TLRs). TLRs are a family of pattern recognition receptors that recognize pathogen-associated and damage-associated molecular patterns. Activation of these receptors is crucial for orchestrating and sustaining the inflammatory response to both types of danger signals. The TLR family consists of 10 known receptors, and there is at least some evidence that each of these are expressed on or within human platelets. This review presents the literature on TLR-mediated platelet activation for each of these receptors, and the existing understanding of platelet-TLR immune modulation. This review also highlights unresolved methodological issues that potentially contribute to some of the discrepancies within the literature, and we also suggest several recommendations to overcome these issues. Current understanding of TLR-mediated platelet responses in influenza, sepsis, transfusion-related injury and cardiovascular disease are discussed, and key outstanding research questions are highlighted. In summary, we provide a resource—a “researcher’s toolkit”—for undertaking further research in the field of platelet-TLR biology.

## 1. Introduction

In the traditional working model of platelet biology, platelets function to localize, amplify and sustain the coagulation cascade at the site of vascular injury [1]. To add another string to their bow, platelets are increasingly appreciated as crucial effectors of leukocyte-mediated immunity [2]. Armed with this skillset, platelets can drive thrombo-inflammation in various clinical conditions, including infection and sepsis [3], cardiovascular disease (CVD) [4], ischemia/reperfusion injury [5], and cancer [6]. Platelets have also emerged as active regulators, rather than simply propagators, of inflammation [7]. Thus, the revised model of platelet biology places these cells firmly at the interface of vascular immunity and thrombosis.

At the nexus of these platelet functions is the expression and activation of Toll-like receptors (TLRs). TLRs are a family of pattern recognition receptors (PRRs) that are widely expressed on immune cells, including platelets, and constitute the first-line of defense against infection and injury [8]. TLRs can recognize both broad microbe-specific pathogen-associated molecular patterns (PAMPs) and host-derived damage-associated molecular patterns (DAMPs), and these receptors are crucial for orchestrating and sustaining the inflammatory response to both types of danger signals.

For platelets, TLR expression and activation elicits a plethora of responses including activation, homotypic and heterotypic aggregation, release of inflammatory mediators and modulation of leukocyte inflammatory responses. Platelets are also key secretors of TLR-triggering DAMPs and, in this capacity, act as initiators of innate immunity. Both the activation of platelet-TLRs and the release of DAMPs have emerged as pathomechanistic in various bacterial and viral infections [9,10,11] and sepsis [12,13] as well as in sterile inflammatory responses including CVD [14,15,16] and transfusion-related adverse clinical reactions [17,18,19]. This review provides an update on platelet-TLRs and their role in infectious and sterile inflammation. We first briefly discuss the complex repertoire of immune responses that platelets can mount during infectious and injurious inflammation, demonstrating their capacity to act as both thrombotic and immune cells.

## 2. Platelets and Their Complex Role in Inflammation

Platelets are complex immune cells, and our understanding of their role in leukocyte-mediated immunity is rapidly expanding. The culmination of the discussion presented below indicates that platelets finely regulate inflammation in a more complex way than has been historically appreciated.

### 2.1. Differential Granule Release

**Figure 1:**
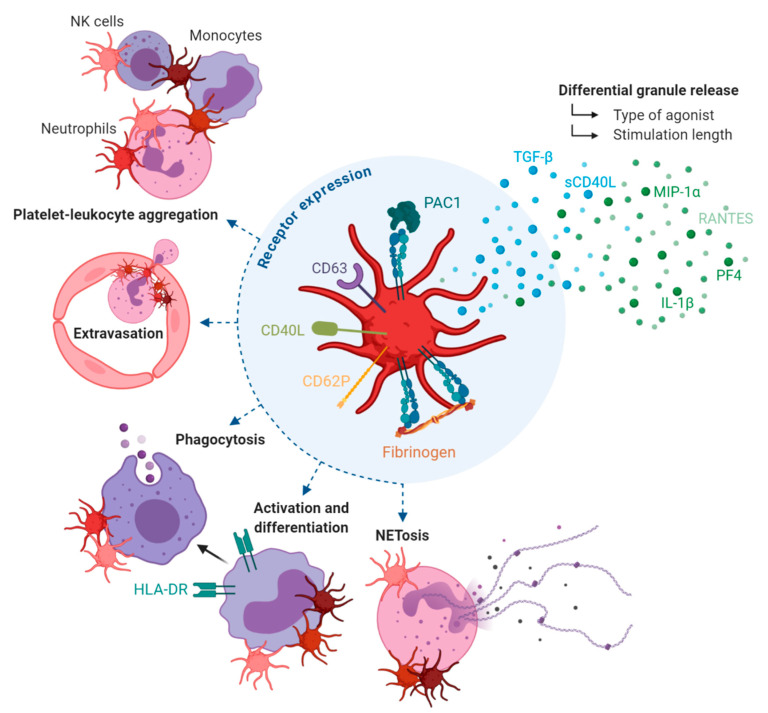
Platelets are complex immune cells that can induce a plethora of inflammatory responses. Platelets activate principally via their differential secretion of granule contents and adhesion receptor expression. Via these mechanisms, platelets can elicit leukocyte-mediated inflammation, including the responses indicated in this figure.

Amongst the factors that can be secreted upon platelet activation, the immunological functions of platelets can be highlighted by their release of numerous cytokines and chemokines. Common factors in platelet releasates include interleukin (IL)-1β, transforming growth factor (TGF)-β, RANTES (regulated upon activation, normal T cell expressed and secreted; CCL5), macrophage inflammatory protein (MIP)-1α, platelet factor 4 (PF4; CXCL4) and soluble CD40 ligand (sCD40L), and also include the release and subsequent surface exposure of receptors such as P-selectin (CD62P), CD63 and CD40L [20] (Figure 1).

Together, these mediators allow platelets to receive and respond to immunologically relevant information in order to steer early inflammatory responses [20,21,22]. For example, in their review on the subject, Semple et al. [22] discuss the dynamics between platelet-derived TGF-β and differentiation of CD4^+^ T cells into regulatory T cells (T_reg_). Platelets are, in large part, responsible for regulating circulating levels of TGF-β which, in turn, is required for the differentiation of T_regs_. The importance of this relationship is highlighted by the observation that T_reg_ numbers and function are impaired in thrombocytopenic disorders [23,24].

Platelet immune-secretory responses are much more complex than previously thought [25]. Granule contents are heterogeneous and show surprisingly little co-localization patterns [26,27,28], indicating distinct packaging of these factors to facilitate diversity in platelet function. Furthermore, distinct platelet agonists or stimulation lengths promote differential release of granule contents, which can induce opposing functions [29,30,31]. For example, Italiano et al. [29] report the segregation of pro- and anti-angiogenic molecules within subsets of platelet α-granules, as well as the distinct release of these factors upon stimulation with different agonists. The dynamics of platelet degranulation invite us to consider platelets as active immune mediators that can induce context-dependent immunological signals, often in a temporal or polarized manner.

### 2.2. Adhesion Receptor Expression

Platelets express several adhesion receptors that are responsible for physically interacting with both endothelial cells and leukocytes, which contributes to both transducing and localizing the inflammatory response. Adherent platelets translocate CD62P from within α-granules to the surface, allowing for the interaction of platelets with leukocytes expressing P-selectin glycoprotein ligand (PGSL)-1. Activated platelets can bind to monocytes, neutrophils, Natural Killer cells, eosinophils and basophils during the course of various inflammatory conditions [32,33,34], thus effectively localizing and linking the innate and adaptive arms of the immune response (Figure 1).

Using intravital microscopy, Zuchtriegel et al. [35] report that acute inflammation promotes the rapid adherence of platelets to endothelial cell junctions through the interaction of platelet glycoprotein (GP) IIb/IIIa (αIIbβ_3_) with von Willebrand Factor (vWF) expressed within these junctions. This step is concomitant to the formation of heterotypic aggregates with neutrophils or inflammatory monocytes. These cell-cell interactions rely on both CD62P-PSGL-1 and CD40-CD40L complexes, possibly in a sequential fashion [35,36], and promote leukocytes to extravasate to the inflammatory site [35]. The requirement for platelet-directed leukocyte extravasation is eloquently described in models of CD62P-deficient mice, where local recruitment of leukocytes is disturbed [37,38]. In such a manner, platelets link the site of inflammation to the immune cell response in which extravasation from the vasculature is a prerequisite.

### 2.3. Leukocyte-Mediated Inflammation

Aside from extravasation, exposure of leukocytes to platelets and platelet factors can induce various cellular responses. PF4 secretion has been shown to drive leukocyte phagocytosis [39], monocyte activation and differentiation [40], neutrophil adhesion [41] and recruitment of both cell types to the endothelium [42] (Figure 1). Platelets are also active participants in inducing neutrophil extracellular trap (NET) formation [43,44,45]. This is a process by which neutrophils expel protein-laced DNA tendrils that form extracellular scaffolds capable of inflammation and thrombosis. In most of these examples, the addition of activated platelets to neutrophil cultures drives NETosis to a greater extent than that seen in the absence of platelets [43,44].

Upon platelet activation, CD40L is massively exposed to the surface and is eventually cleaved into its soluble form (sCD40L) [46,47]. CD40L expressed on the platelet surface is able to induce dendritic cell maturation, B-cell isotype switching and potentiate the function of CD8+ T cells [48,49,50,51], thus linking important innate and adaptive immune responses. sCD40L can also simultaneously link different receptors, increasing the potential contexts in which this ligand may be involved in after secretion by platelets [48,52]. For example, although with less affinity, CD40L can also bind to GPIIb/IIIa on the surface of platelets [51], which is likely to amplify platelet-platelet communication.

### 2.4. Platelets also Regulate Leukocyte-Mediated Inflammation

In addition to their immune-stimulatory role, there is growing evidence that platelets are important immunoregulators that provide anti-inflammatory cues to limit host damage [7,53]. This is often elicited at the platelet: leukocyte interface [54,55,56,57]. To date, this anti-inflammatory platelet effect has been mostly observed as a change in the cytokine secretion profile by PBMCs. For example, pre-activated platelets, either with thrombin receptor activator peptide (TRAP) [54] or with autologous thrombin (to form a platelet gel) [55], significantly reduced the release of pro-inflammatory cytokines (for example, tumor necrosis factor (TNF)-α) and increased the release of anti-inflammatory cytokines (IL-10) by PBMCs. As with PBMCs, pre-treatment with platelets can reduce ROS production by stimulated neutrophils [58,59], an effect that can be reversed by administration of anti-platelet therapy [58].

Platelets are thought to exert these effects when the inflammatory response becomes excessive and unnecessary. For example, neutrophil-mediated inflammation is necessary for host defense but, as a by-product, can often cause host damage [60,61]. There are many examples where platelets can keep neutrophilic inflammation in check [58,59,62] to regulate the extent of this damage. In an example of the juxtaposition of their roles in inflammation, platelets aggregate with neutrophils to propagate vascular damage in acute lung injury [63,64] but these cells can also temporally produces pro-resolving mediators to reduce lung inflammation [65]. It is now appreciated that platelets can have both pro-and anti-inflammatory effects, perhaps in a context-specific [66,67] and sequential [65,68] manner.

## 3. TLRs

Platelets are known to induce the range of inflammatory functions described above via activation of their TLRs. TLRs are a family of PRRs that are constitutively expressed on immune cells and act as sentinels against both exogenous and endogenous ‘danger’ signals. These receptors are a first-line defense against both DAMPs and PAMPs and set in motion a series of molecular events that leads to initiation of innate immunity.

Within the mammalian TLR family, there are 10 identified receptors which can be divided into two subpopulations by their cellular localization and, hence, by their PAMP/DAMP specificity. TLR1, 2, 4, 5, 6 and 10 are expressed on the cell-surface and traditionally respond to extracellular lipid molecular patterns. TLR3, 7, 8 and 9 are expressed in intracellular compartments of immune cells and traditionally detect microbial nucleic acid components that are by-products of microbial digestion. Most TLRs transduce their signal via homodimerization following ligand binding. However, TLR2 is unique: this receptor will heterodimerize with either TLR1 or TLR6 to recognize different bacterial lipopeptides (tri-acylated and diacylated lipopeptides, respectively).

In nucleated cells, TLRs induce innate immune responses via activation and nuclear translocation of nuclear factor-κB (NF-κB) and subsequent production of NF-κB-controlled pro-inflammatory cytokines [69]. NF-κB can be considered a ‘master controller’ of inflammation and, in activating this transcription factor, TLRs rapidly induce inflammation in response to PAMP/DAMP ligation. In anucleate platelets, TLR signaling transduction is fundamentally different, and involves numerous platelet activation pathways [70,71] including a non-genomic role for NF-κB activation [72,73]. Indeed, NF-κB signaling is crucial for inducing a range of platelet functions including PAC-1 and fibrinogen binding, aggregation, and adenosine triphosphate (ATP) release in response to both thrombotic [74] and inflammatory [72] stimuli.

We provide this brief discussion of TLRs here as a preface to discussing, in more detail, the role of platelet-TLRs in the section below. For greater understanding of the signaling and function of this receptor family, we direct the reader to a number of comprehensive reviews on these topics [8,75,76].

## 4. An Update on Platelet-TLR Expression and TLR-Mediated Platelet Activation

Since the first reports of their presence on and within platelets [77,78], the role of platelet-TLRs has been extensively studied, although with a favor towards a subset of these receptors. This section provides an update on each platelet-TLR, and their involvement in human infection and sterile inflammation. Throughout this section, we make mention of several methodological considerations that, if adopted by future studies, aim to resolve some of the conflicting data discussed herein. These considerations are also described in detail in Table S1. We also refer to several supplementary tables (Tables S2–S6) that comprehensively documents the studies described in this section. The culmination of these tables, and this section, serves as a “researcher’s toolkit” to aid in examining platelet-TLR expression and activation in future studies.

### 4.1. TLR2 and Its Binding Partners, TLRs 1 and 6

#### 4.1.1. Expression of Platelet-TLRs 1, 2 and 6 in Health and Disease

**Figure 2:**
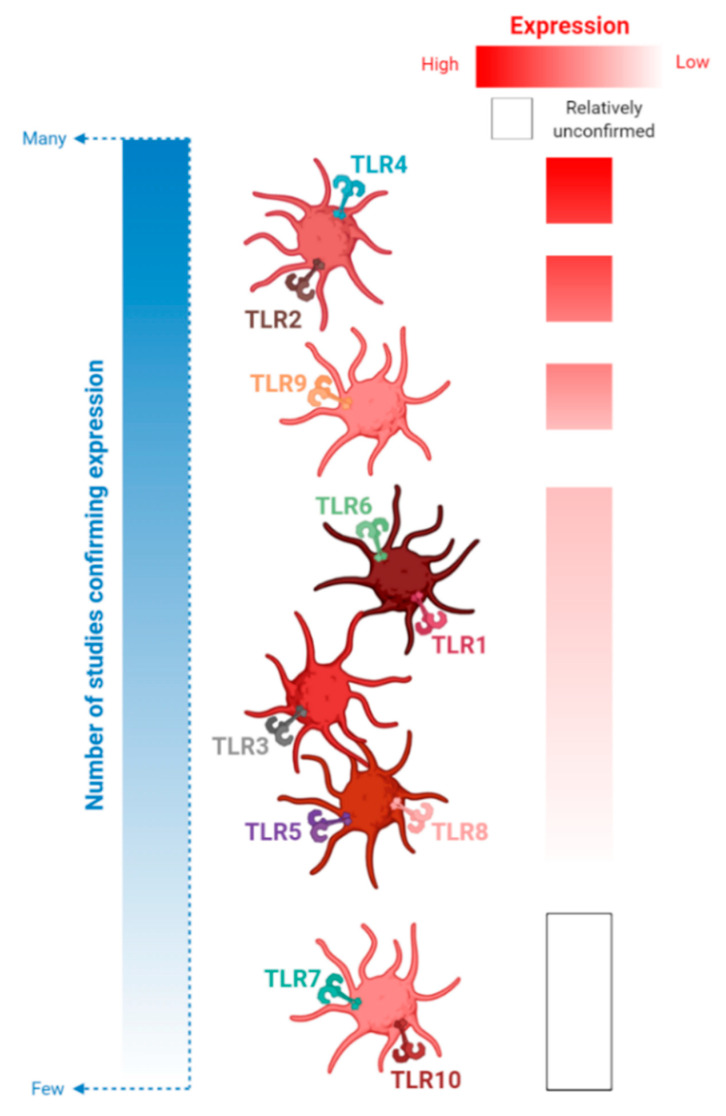
A summary of platelet-TLR expression. All 10 TLRs have been identified on and within human platelets. In this figure, platelet-TLRs are stratified by the number of studies that have measured expression levels (many vs. few), as indicated by the left-hand blue panel. Platelet-TLRs 2 and 4 are the most well-studied while only a few papers investigate expression of platelet-TLRs 7 and 10. The extent of expression on and within platelets is indicated by the right-hand red panel. Most platelet-TLRs (TLRs 1, 3, 5, 6 and 8) are expressed at low levels while others (TLRs 2, 4 and 9) are expressed more abundantly. Due to the low number of studies investigating TLRs 7 and 10, it is difficult to determine the extent of their expression on/within platelets.

TLRs 1, 2 and 6 expression on the platelet surface has been confirmed at both the protein [16,71,78] and mRNA [79] level. All three TLRs are expressed predominantly on the platelet surface, but also intracellularly. While TLR2 is moderately expressed, TLRs 1 and 6 are expressed at relatively low levels (Figure 2 and Table S2).

Furthermore, platelet-TLR2 expression has been shown to increase in patients with acute coronary syndromes (ACS) at both the protein [14] and mRNA [15] level, compared to controls with normal coronary arteries. Similarly, a significant increase in platelet-TLRs 1 and 6 was also reported in patients with ACS [16]. In addition, platelets sampled from both the periphery and the left atrium had significantly higher levels of surface TLR2 expression in patients with atrial fibrillation (AF) compared to those without AF [80]. Platelet-TLR2 expression was also higher in those with persistent, as opposed to paroxysmal, AF. These results suggest that upregulated platelet-TLR expression correlates with the heightened thrombo-inflammatory state during both ACS and AF.

Interestingly, no change in cell-surface expression of platelet-TLR2 was observed in septic patients, compared with both healthy and non-infectious controls within the intensive care unit [81] and this is despite infection inducing a similar magnitude of acute inflammatory response as that seen in ACS. Similarly, compared to healthy controls, no augmentation in platelet-TLR2 expression was seen in patients with essential thrombocythemia, a clinical condition associated with sustained thrombocytosis and thrombo-inflammation [82]. Platelet-TLR2 seems to be regulated variably during inflammation and seems likely to be important for the inflammatory response to cardiovascular pathology.

#### 4.1.2. TLR2-Dependent Bacterial and Viral Activation

Platelets are active participants in hemostatic and immune surveillance, and the “en garde” nature of these cells ensures their rapid deployment and activation during bacteremia and viremia. TLR2-mediated signaling during periodontal bacteremia plays a central role in platelet activation [83] and systemic inflammation [84], and is also considered a risk factor for cardiovascular disease [85]. Understanding the mechanisms by which these pathogens sustain bacteremia is clinically important, and the role of platelet-TLR2 in this context has been extensively studied.

Assinger and colleagues [10,11] have demonstrated that distinct profiles of platelet activation are triggered in response to in vitro periodontopathogenic infection, compared to “traditional” thrombotic stimulation. Stimulation with two representative periodontopathogens (*Porphyromonas gingivalis* and *Aggregatibacter actinomycetemcomitans*) induced significant platelet-neutrophil aggregation [11,70], and this interaction with platelets significantly enhanced bacterial binding and phagocytosis by neutrophils [11]. Heterotypic aggregation in response to both bacterial species was reduced by TLR2 blockade more effectively than by TLR4 blockade but, interestingly, simultaneous inhibition of both TLRs had a synergistic effect on platelet-neutrophil interactions [11].

Both periodontal strains also induced platelet-surface CD63 [11] and CD40L [10] expression and these responses were also reduced by TLR2 blockade and, to a lesser extent, TLR4 blockade. Platelet activation and platelet-neutrophil aggregation in response to periodontopathogens contrast with responses to ‘traditional’ platelet agonists: stimulation with TRAP and adenosine diphosphate (ADP) can induce greater platelet activation but reduced heterotypic aggregation, compared to periodontopathogenic stimulation [11].

While there is a clear link to the involvement of both platelets and TLR2 in these studies, the role of TLR2 in mediating platelet responses to *Streptococcus pneumoniae* is not as well defined. *S. pneumoniae* can induce platelet aggregation and ADP release in both a TLR2-dependent [86] and TLR2 (and TLR4)-independent [87] manner. Keane and colleagues describe *S. pneumoniae*-induced platelet aggregation as a strain-specific “all-or-nothing” response: strains either elicited rapid and robust aggregation, or platelets did not respond [86]. The authors also show that pre-incubation with a TLR2 antibody completely abrogated platelet aggregation with a representative *S. pneumoniae* strain [86]. In a similar experimental design, Liu and colleagues also demonstrate that platelet aggregation induced by *Streptococcus agalacticae* was strain-specific and completely dependent on platelet-TLR2 signal transduction [88]. de Stoppelaar and colleagues [87] also demonstrated platelet aggregation in response to some *S. pneumoniae* serotypes, but this response and others (platelet surface CD62P and CD63 expression) were not changed in the presence of TLR2 and TLR4 blockade. The reason for the differences in response to *S. pneumoniae* is unknown but suggests that platelet-TLR2 activation is strain-specific and likely to vary with bacterial culture methods and bacterial load used in platelet activation assays.

Human cytomegalovirus (HCMV) is another pathogen that can establish chronic inflammation, and infection and re-activation are associated with heightened cardiovascular risk [89]. HCMV specifically binds to the sub-population of platelets that express TLR2 and can induce platelet-leukocyte aggregation in both a CD62P and TLR2-dependent manner [9]. Furthermore, platelets can enhance HCMV-mediated neutrophil activation, leukocyte adhesion and transmigration while HCMV fails to induce platelet pro-thrombotic responses, including platelet aggregation [9]. This latter result strengthens the notion that platelet-TLR2 activation is preferentially involved in immune, rather than thrombotic, responses to bacterial or viral infection.

**Figure 3:**
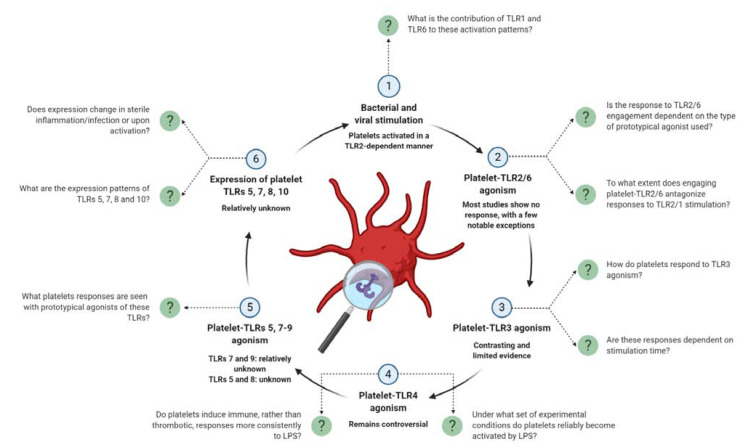
Unanswered questions that remain in the field of platelet-TLR biology.

It is interesting to note that the extent to which TLRs 1 and 6, which heterodimerize with TLR2 for signal transduction, contribute to these platelet responses remains an unanswered question (Figure 3).

#### 4.1.3. TLR2/1 Engagement with Pam3CSK4

**Figure 4:**
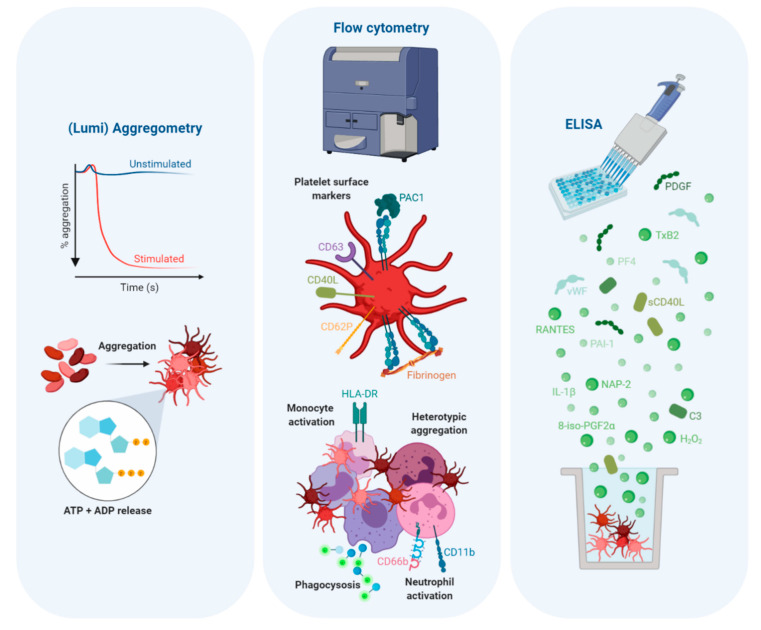
A summary of the techniques and associated measurements that are most commonly used for assessing platelet responses to TLR agonism.

Of the literature examining platelet-TLR agonism, the most solid conclusion that can be drawn is that platelets are directly activated by the prototypical TLR2/1 agonist, Pam3CSK4. Pam3CSK4 is used exclusively for assessing platelet-TLR2/1 engagement. With a few notable exceptions, this agonist can stimulate a range of thrombotic (fibrinogen and PAC1 binding, aggregation, ATP and ADP release) and immune (platelet-leukocyte aggregation, release of inflammatory mediators) responses (see Table S3 for all references). These responses are amongst the most common measurements used when examining platelet-TLR function. A graphical summary of these common measurements is shown in Figure 4.

#### 4.1.4. Signaling Pathways Involved in Pam3CSK4-Mediated Platelet Activation

The existence of the diverse multicomponent NF-ĸB family in platelets indicates that, depending on the source of platelet stimulation, different associations of NF-κB subunits can induce distinct signaling pathways [90,91]. Several studies have demonstrated the requirement for non-genomic NF-ĸB activation in response to Pam3CSK4 [72,92]. This signaling pathway is activated at a much slower rate than that seen with TRAP stimulation [92], indicating that thrombotic and immune stimulation utilize similar signaling molecules, but in distinctly different ways. In other studies, in vitro Pam3CSK4-mediated platelet responses were shown to depend on the sequential release of ATP and ADP and ligation to their platelet purinergic receptors P2X_1_ (ATP), P2Y_1_ and P2Y_12_ (ADP), as well as the generation of the cyclooxygenase metabolite, thromboxane A_2_ (TxA_2_) [93,94]. A comparable requirement for ADP and ATP release was shown for HCMV-mediated platelet activation via TLR2 [9].

ATP/ADP release constitute important pathways that can amplify the initial platelet response to TLR2/1 engagement. Given the relatively low surface expression of TLR1 and moderate expression of TLR2 [70,77,95,96] there is likely to be a low potential for Pam3CSK4 ligation on the surface of resting platelets. Amplification via these pathways may be a mechanism to ensure robust TLR2/1-mediated activation. Another mechanism of these amplification pathways may be to enhance platelet-surface TLR2 expression [81,97] to ensure sufficient exposure and ligation to TLR2 agonists.

Interestingly, oral administration of both ticagrelor (to inhibit ADP:P2Y_12_) and aspirin (to inhibit TxA_2_ production) to healthy volunteers did not show any reduction in Pam3CSK4-mediated platelet activation at most doses tested [98]. Significant, but modest, reduction in platelet activation was only noted at a supra-physiological dose (100 µg/mL) [98]. It may be that, despite the requirement for amplification of Pam3CSK4-mediated platelet activation by ADP and TxA_2_, oral administration of anti-platelet therapy is unable to sufficiently inhibit these amplification pathways. This has implications for treatment of conditions such as ACS. Dual anti-platelet therapy (DAPT; a combination of aspirin and a P2Y_12_ receptor antagonist) is the mainstay of therapeutic intervention to prevent platelet activation post-ACS, but platelet-TLR2/1 remains a functional on-treatment activation pathway in these patients [16,98,99].

#### 4.1.5. Platelet-TLR2/1 Engagement and Immunity

Similar to the description of periodontopathogenic stimulation, Rex et al. [100] showed that Pam3CSK4-stimulated platelets aggregate to a lesser extent than in response to thrombin. However, stimulation with this agonist causes significant platelet-monocyte aggregation that was not seen with thrombin [100]. Platelets also significantly aggregate with both neutrophils and monocytes upon TLR2/1 engagement [11,16,56,72,100,101] and a number of studies have investigated the consequences of platelet co-culture on leukocyte responses to Pam3CSK4. Carestia et al. [44] demonstrate that the presence of platelets can enhance Pam3CSK4-mediated NET formation in a TLR2-dependent manner. These authors suggest that this enhancement was dependent on vWF release and subsequent bridging between platelet-GPIb and neutrophil-CD18 expression, as antibody blockade of both cell-surface receptors abrogated NETosis. Platelets likewise can enhance Pam3CSK4-mediated myeloperoxidase (MPO) release [101] and phagocytosis [66] by neutrophils.

However, these responses are juxtaposed with the role of platelets in dampening the release of inflammatory cytokines from peripheral blood mononuclear cells (PBMCs) [56] as well as dampening neutrophil activation and elastase secretion [66,67] in response to Pam3CSK4. The reduction in PBMC cytokine production was observed by Tunjungputri et al. [56] to be platelet concentration-dependent, and dependent on both platelet-monocyte aggregation and phagocytosis of platelets by monocytes. Oral administration of ticagrelor reversed this platelet effect: TLR2/1-mediated production of pro-inflammatory cytokines in whole blood increased with ticagrelor, perhaps due to the reduced ability of platelets to complex with monocytes [56]. In these studies, the platelet effect is modest. For example, platelets can reduce the expression of neutrophil activation markers by approximately 15% [66,67]. A similar regulation, rather than abrogation, of PBMC responses by platelets in response to TLR2 has been demonstrated [56,67]. In this capacity, platelets may act as a, perhaps temporal, brake to leukocyte inflammation in order to prevent rampant host damage [7].

#### 4.1.6. TLR2/6 Engagement

Despite ample corroboration that platelets respond directly to TLR2/1 agonism, we lack the same base of evidence for the platelet-TLR2/6 response. In most studies, stimulation with prototypical TLR2/6 agonists is unable to induce platelet aggregation [70,87,93], activation or heterotypic aggregation [16,87] (Table S3). Interestingly, despite demonstrating that macrophage activating lipopeptide-2 (MALP-2) cannot induce platelet activation, pre-incubation with this TLR2/6 agonist dose-dependently antagonized the effects of TLR2/1 engagement with Pam3CSK4 [93]. This is an interesting finding and has not been investigated beyond this study. Importantly, in acting as a receptor antagonist for TLR2/1, MALP-2 may attenuate TLR2/1-mediated platelet activation during sepsis and other clinical conditions that are a potent source of TLR-triggering ligands. The extent of this antagonism effect, and whether this extends to other TLR2/6 agonists, is worth exploring further (Figure 3).

At supra-physiological doses, fibroblast stimulating lipopeptide-1 (FSL-1), another prototypical TLR2/6 agonist, induced platelet activation and heterotypic aggregation in whole blood but not in platelet-rich plasma (PRP) [16]. This may suggest an indirect role for platelet TLR2/6 engagement by activation of TLR-expressing leukocytes. Furthermore, in a mixed cell culture model, the presence of platelets modulated both neutrophil and PBMC responses to FSL-1 [66,67]. With FSL-1 stimulation, platelet co-culture significantly reduced neutrophil activation and elastase release [66,67] but also increased neutrophil phagocytosis [66]. In a similar complex manner, platelets reduced PBMC production of TNF-α, but also increased the production of IL-10, MIP-1β and IL-6 in response to FSL-1 [67]. Platelets are shown here to attenuate and simultaneously augment leukocyte function in response to TLR2/6 engagement, potentially as a sequential protective mechanism against inflammatory host damage.

In another example of alternative activation of TLR2/6, Biswas et al. [96] demonstrated that a specific group of oxidized phospholipids (oxPC) that signal via the PRR, CD36 (oxPC_CD36_), activate platelets in a CD36/TLR2/6-dependent manner. The authors suggest that heightened platelet reactivity during hyperlipidemia may be linked to increased ligation of oxPC_CD36_ to platelet-TLR2/6. In addition, the authors also demonstrate that platelet CD62P expression increased with Pam2CSK4 stimulation (another TLR2/6 agonist), a response which was abrogated by TLR6 antibody blockade [96]. This resonates with Blair et al. [70], who report platelet aggregation, albeit inconsistent, in response to the same TLR2/6 agonist. However, these results from Biswas et al. [96] and Blair et al. [70] are in contrast to a number of other studies that show no response to platelet-TLR2/6 stimulation [16,87,93].

Potentially, some TLR2/6 agonists (Pam2CSK4) may have greater potency than others (MALP-2, FSL-1) for direct platelet activation and aggregation. When examining platelet responses to TLR2/6 (and other TLRs) engagement, a range of prototypical agonists exist although the choice of agonist varies greatly between studies. This perhaps suggests a need to examine the effects of more than one TLR agonist for future research (Figure 3, Table S1).

### 4.2. TLR3

In contrast to TLR2, the role of platelet-TLR3 remains unclear and understudied. Platelets are known to express very low cell-surface and moderate intracellular expression of TLR3 [102,103] (Figure 2 and Table S4), although agonizing platelet-TLR3 seems to have contrasting results (Table S5).

More specifically, Anabel et al. [102] show that platelets immediately mobilize intracellular calcium upon stimulation with the prototypical TLR3 agonist, poly(I:C). Over longer stimulation periods, platelets release PF4 (30 min stimulation) and IL-1β (3 to 16 h). poly(I:C) also increases platelet surface CD62P expression (60 min). However, D’Atri et al. [103] show that both poly(I:C) and poly(A:U) potentiated platelet aggregation, ATP release and fibrinogen binding over a much shorter time frame (6 min stimulation), although these agonist were not able to directly induce these responses. Furthermore, platelets were unable to induce CD62P or CD40L expression with either TLR3 agonist alone, and no potentiation of thrombin-induced expression of these markers was observed (15 min) [103].

It may be that platelets require priming or a combination of agonists to immediately aggregate via TLR3 but can become activated by TLR3 agonists alone with longer stimulation times. Like that seen with TLR2, it may also be that platelets preferentially induce immune activation (for example, IL-1β release) over thrombosis. Many of these immune functions occur, and are therefore analyzed, over longer stimulation times than traditionally seen with thrombotic measurements. Assessing a range of platelet functions (immune and thrombotic) and optimizing the stimulation time for each of these functions, are important parameters to consider for future research (Table S1). As with responses to TLR2/6 engagement, an update on platelet-TLR3 agonism is required to add to this discussion (Figure 3).

### 4.3. TLR4

#### 4.3.1. Expression of Platelet-TLR4 in Health and Disease

Platelets abundantly express surface TLR4 [77,87,95,97,104,105] (Figure 2 and Table S2), and platelet-TLR expression has also been shown to increase in patients with ACS [14,16], sepsis [12], community-acquired pneumonia [106] and is modulated during Crohn’s disease during the acute and remission stages [107]. In the study of sepsis, upregulation of platelet-TLR4 was most evident on admission and persisted until day 5 of hospitalization [12]. Similarly to the results described for platelet-TLR2 expression, platelets express TLR4 at a significantly higher level in patients with AF compared to non-AF controls [80]. Within the AF cohort, the greater time spent in AF (persistent vs. paroxysmal) was associated with an increase in platelet-TLR4 expression [80]. In contrast, no modulation of platelet-TLR4 expression is seen in patients with Essential Thrombocythemia, compared to healthy controls [82]. However, the culmination of these results suggests that platelet-TLR4 is actively upregulated during most clinical forms of infectious and injurious inflammation.

#### 4.3.2. Engaging Platelet-TLR4 with Lipopolysaccharide (LPS)

Despite the large body of work that examines the role of platelet-TLR4, whether LPS can robustly activate platelets remains contested. There is a consensus that, although LPS cannot cause platelet aggregation, this prototypical TLR4 agonist can potentiate aggregation with sub-threshold concentrations of traditional thrombotic agonists (see Table S6 for all references). Initial LPS binding to platelets is mediated by TLR4 but likely requires the subsequent upregulation of cell-surface CD62P, as LPS binding can be reduced with polyclonal CD62P blockade [105,108]. This may explain why potentiation with thrombotic agonists is required to see an effect of LPS stimulation—the addition of a second agonist allows for upregulated CD62P to sustain a response to LPS. Indeed, TLR4 and CD62P co-localize on the surface of thrombin-activated platelets [105].

A confusing picture emerges when examining whether LPS can elicit the expression of platelet-surface activation markers. For example, LPS has been reported to cause and/or potentiate platelet-fibrinogen binding in some [72,105,109] but not other [110] studies. Similar observations have been made for CD63 (expressed [111,112] vs. not expressed [81,87]), PAC1 ([105] vs. [16]) and CD62P ([113] vs. [43,81,87,110,113,114,115]). In one study, platelet activation was only seen in response to supra-physiological doses of LPS (100 μg/mL) [67] but not in response to lower doses (0.1 to 50 μg/mL).

In general agreement with platelet-TLR2 responses, LPS may have a greater effect in initiating direct immune, rather than thrombotic, responses. For example, LPS can consistently and directly induce the release of a number of inflammatory mediators including IL-1β [116], sCD40L [10,111], RANTES [97], and can potentiate the production of eicosanoids and oxidative stress markers [106]. Interestingly, platelets were shown to induce [43] and potentiate [44] NETosis in response to LPS, and this interaction increases bacterial trapping by neutrophils [43]. However, this latter result contrasts with a key finding from other studies. This LPS-mediated NETosis was reliant on a strong interaction of platelets with neutrophils [43]. Although this has been observed by some [11], a majority of other studies have reported no heterotypic aggregation in response to LPS [16,56,81,87].

The effect of platelets on PBMC responses to LPS is more complex and contrasting. In one study, the presence of platelets was shown to induce a pro-inflammatory environment (increased IL-1β and IL-6; reduced IL-10) [56]. However, in a similar experimental design, LPS was also shown to reduce inflammation (reduced IL-6, TNFα and MIP-1β; increased IL-10) [67]. In this latter study [67], and in a follow-up study [66], platelets could also reduce both neutrophil and monocyte activation in response to LPS, to varying degrees. In a similar study, platelets that were introduced to high shear stress were able to significantly induce IL-10 production from immature dendritic cells stimulated with LPS and IFN-γ [117]. In this study, Hagihara et al. [117] recognized that blood flow-induced mechanical forces are physiological activators of platelets, and that platelets are sensitive to shear stress in circulation. Using shear-based, alongside static-based, conditions may provide a more physiologically relevant experimental set-up for examining platelet-TLR responses. Mimicking shear stress experimentally may also help to understand how agonist-induced platelet responses change under more complex, pathological hemodynamics by comparing conventional versus high shear conditions. However, examining shear stress requires specialist equipment that may have restricted the widespread examination of shear-induced platelet-TLR responses thus far. As discussed previously, these contrasting results support the notion that platelets can both promote [118,119] and dampen [54,55] leukocyte-mediated inflammation.

Investigation into the effectiveness of a number of P2Y_12_ receptor antagonists have supported the notion that P2Y_12_ antagonism only modestly modulates TLR4-mediated platelet activation. For example, prasugrel did not reduce LPS-mediated sCD40L and sCD62P levels in a human endotoxemia model [120]. In a similar manner, DAPT (aspirin and ticagrelor) was only able to modestly reduce platelet activation in response to LPS at the highest dose (100 µg/mL) tested [98].

As an alternative to TLR4 engagement with LPS, the addition of platelet microparticles (MPs) to platelets was shown to induce various platelet responses in a partial TLR4-dependent manner [121]. Co-incubation with MPs significantly increased both heterotypic and homotypic aggregation and accelerated clotting. Platelet-neutrophil aggregation in the presence of MPs was completely abrogated with TLR4 blockade while platelet aggregation and clotting parameters were modestly, but significantly, reduced. Again, these results support that engaging TLR4 pushes platelets towards an immune activation profile and strengthens the argument that most platelet-TLRs act in this capacity. Perhaps the focus of future studies should be on interrogating the immune (rather than thrombotic) responses induced by platelet-TLR4 ligation (Figure 3).

#### 4.3.3. Methodological Considerations for Examining Platelet Responses to LPS

The variability in platelet responses to LPS may be due to the dose range tested. Most studies have examined the effect of between 1 and 10 µg/mL of LPS [43,44,72,81,87,105,109,110,111,114,115]. It is difficult to accurately ascertain the circulating physiological levels of these agonists and, therefore choose a dose to test in vitro. To complicate the matter, TLR ligands may accumulate at sites of infection or injury to form a highly localized dose that platelets, and other cell types, are exposed to. One such example is the accumulation of LPS in atherosclerotic lesions [122]. Nocella et al. [106] demonstrated that circulating plasma and serum levels of LPS endotoxin were 107 and 145 pg/mL, respectively, for patients with community-acquired pneumonia. These authors were also able to demonstrate that a dose as low as 15 pg/mL was able to potentiate platelet responses [106]. In a nod to the role of platelet-TLRs in inducing and regulating immunity, the release of IL-1β [116] or the regulation of leukocyte responses [56,66,67] is often examined with much lower doses of LPS (ng/mL vs. μg/mL) and over much longer stimulation times. Both the dose range and length of stimulation are important considerations here (Figure 3 and Table S1).

Such inconsistency in platelet-TLR4 responses is also likely due to the use of various chemotypes and preparations of LPS used. Within studies, the potency of different LPS chemotypes varies in the ability to bind to platelets [105], induce cyclic guanosine monophosphate (cGMP) production [123] and induce [112] or potentiate various measures of platelet activation [109]. For example, stimulation of platelets with either *Escherichia coli* O111 LPS or *Salmonella minnesota* LPS induced the release of supernatants that triggered differential IL-6, TNFα, and IL-8 secretion by PBMCs [112]. In a similar vein, Kappelmayer et al. [114] describe that, although both LPS forms are biologically active, the rough form of LPS (Re-LPS) but not smooth LPS (S-LPS) from *E. coli* directly modulated platelet activation. Recently, Vallance et al. [110] demonstrated that the use of three ultrapure serotypes under a range of experimental conditions (for example, room temperature vs. 37 °C, various stimulation times) was not able to robustly induce platelet responses [110]. In agreement to an earlier observation made for future examinations of platelet-TLR2/6 agonism, the use of more than one source of LPS to investigate the platelet response is warranted in this context (Figure 3 and Table S1).

A number of studies, with seemingly contrasting results, have highlighted the importance of the platelet preparation for eliciting responses to LPS. With LPS, upregulation of CD63 on the platelet surface [111,112] and sCD40L release [111] was shown to be greater in PRP than in washed platelets (WPs), and this response could be partially restored with the addition of CD14 to WPs. In opposition, another study has shown that platelet release of inflammatory mediators in response to direct LPS stimulation was more pronounced in WPs than in PRP [97]. These results directly demonstrate the need to compare multiple types of preparations (WPs vs. PRP vs. whole blood) for a more globalized view of the platelet response to LPS (Figure 3 and Table S1).

### 4.4. TLR7

Similar to TLR3, responses to ligation of platelet-TLR7 has been understudied in the past (Table S5), and this requires addressing in future research (Figure 3). TLR7 is expressed in platelets at both the protein [71] and mRNA level [79,100] (Figure 2 and Table S4). Interestingly, platelet-TLR7 mRNA is reported to be variably expressed by Koupenova et al., with reports of 25% [79] and 60% [100] of human participants positive for this transcript in two large community-based sub-studies of the Framingham Heart Study.

Recent evidence has demonstrated the potential role of this TLR in viral host defense [100,101]. Koupenova et al. [100] were the first to demonstrate that platelets respond to TLR7 agonism by mobilizing TLR7 from within intracellular acidic vesicles and aggregating with neutrophils. Heterotypic aggregation was initiated by platelets and is likely mediated by increased platelet CD62P and CD40L expression. In contrast, platelets were unable to induce a thrombotic response to two TLR7 agonists (loxiribine and R837), strengthening the notion that platelet-TLR engagement tends to more strongly induce immune responses.

Whether or not the platelet-TLR7 transcript is present within an individual is of potential importance for their responses to the influenza virus [101]. Release of complement 3 (C3) by healthy platelets in response to both the influenza A viral strain and the TLR7 agonist, loxoribine, was only evident in those expressing the TLR7 transcript [101]. Agonism of platelet-TLR7, but not neutrophil-TLR7, was shown to specifically induce release of MPO and extracellular DNA from neutrophils and this process was dependent on the release of C3 from platelets [101]. This TLR7-C3 axis provides a mechanism for the involvement of platelets in eliciting neutrophil host defense mechanisms in response to influenza infection.

### 4.5. TLR9

In platelets, TLR9 localizes to membrane-encapsulated regions adjacent to the plasma membrane [124], giving a punctuate and granular intracellular expression pattern [71,124] (Table S4). Thon and colleagues [124] highlighted a novel packaging and distribution mechanism within platelets. The authors show that stimulation with a range of thrombotic agonists translocates TLR9 from intracellular vesicles by vesicle-associated membrane protein (VAMP)7 and VAMP8. The subsequent increase in distribution of TLR9 along the platelet surface potentiates the immediate capture and eventual endocytosis of CpG DNA, the ligand for TLR9 [124]. Interestingly, the host defense mechanism proposed for platelets is in opposition to the response of nucleated cells. For these cells, pathogenic internalization stimulates the distribution of nucleic acids to intracellular vesicles for eventual ligation and activation of intracellular TLR9 (as well as TLRs 3, 7 and 8). This alternative activation of TLR9 (platelet-surface expression) is supported by consistent reports of elevated platelet surface TLR9 expression with thrombotic stimulation [71,77,81,95,124]. Priming of platelets in this way alerts these sentinel cells to the presence of pathogens, and results in pathogenic sequestration and removal from the bloodstream.

In a further study investigating the kinetics of TLR9 activation, Panigrahi and colleagues [71] demonstrate that carboxy(alkylpyrrole) protein adducts (CAPs), end-products of lipid oxidation, are a novel ligand for platelet-TLR9. CAPs are commonly recognized as pathological by PRRs, and also co-immunoprecipitated with platelet-TLR9 in this study. Stimulation with CAPs induced CD62P expression and PAC1 binding and potentiated TRAP- and ADP-mediated platelet aggregation (Table S5) [71]. Furthermore, all responses were diminished with TLR9 antibody blockade [71]. In accordance with Thon and colleagues [124], CAPs may interact with platelet surface TLR9. Once ligated, CAPs upregulate TLR9 expression by mobilizing intracellular TLR9 [71] and eventually amplifies CAP sequestration and platelet reactivity. The authors suggest that such heightened reactivity is an important clinical problem in conditions characterized by oxidative stress (and subsequent production of CAPs), such as hyperlipidemia and atherosclerosis.

### 4.6. TLRs 5, 8 and 10

Platelet-TLRs 5, 8 and 10 remain the most under-studied of this receptor family. The presence of platelet mRNA transcripts for TLRs 5, 8 and 10 was first demonstrated by Koupenova et al. [79] in a large cohort of participants of the Framingham Heart Study (Table S4). Platelet-TLR8 mRNA was expressed in a minority (approximately 15%) of participants, while TLR5 and TLR10 transcripts were expressed in most of the cohort (80 to 85%). Each of these platelet-TLR transcripts were associated with various cardiovascular risk factors stratified by sex, including total cholesterol-to-high-density lipoprotein ratio (TLR5 and TLR10 in women), body mass index (TLR8 in both sexes) and hypertensive treatment (TLR8 in men). Low platelet expression of TLR5 (cell-surface [81]) and TLR8 (cell-surface and intracellular [77]) protein expression has been confirmed and, in the case of platelet-TLR5 expression, was shown to increase in TRAP-stimulated platelets [81].

Interestingly, a recent paper has demonstrated a role for platelet-TLR8 in responding to *Candida albicans*. The authors suggest that this fungal species can reduce platelet reactivity via TLR8 and potentially escape platelet-mediated host defense during candidemia [125]. 

The biological function, beyond being a PRR, and ligand specificity for TLR10 are currently unknown. However, a number of commercially available antibodies exist to determine platelet-TLR10 expression and under which conditions TLR10 expression changes (Figure 3). Similarly, shedding light on platelet activation in response to TLRs 5 and 8 engagement is important, yet unexplored, research (Figure 3).

## 5. Platelets are Capable of Releasing TLR-Stimulating DAMPs

Aside from the role of platelet-TLRs described above, platelets are known to be an important source of DAMPs with the ability to ligate with TLRs. Here, we describe the immune effects of mitochondrial DNA (ntDNA) and High Mobility Group Box 1 (HMGB1), both of which can be released by platelets and cause wide-spread TLR-mediated inflammatory responses.

### 5.1. mtDNA

There is convincing evidence that endogenous mitochondrial DAMPs, including fragments of mtDNA and other mitochondrial-associated proteins, are potent immune-stimulators that can cause human disease [126,127]. The structure and pro-inflammatory effects of mtDNA are similar, if not identical, to bacterial DNA due to its proteobacterial origin. This similarity extends to the ability of mtDNA to stimulate TLR9 [128,129]: mtDNA possesses unmethylated CpG dinucleotides, which is the known ligand for this TLR. Mitochondrial DAMPs play a key role in the activity of local and peripheral immunity. For example, mtDNA is an important requirement for NLRP3 inflammasome activation [130], which occurs after NLRP3 priming via TLR ligation. Likewise, mtDNA can stimulate neutrophil-mediated inflammation and NET formation [17], both processes of which are known to be facilitated by platelets and their TLRs [43,66,101]. mtDNA can also induce endothelial cell permeability, thereby facilitating the transfer of localized the immunogenic response to distal organs [131].

### 5.2. HMGB1

HMGB1 is another well-described DAMP that is released passively by both stressed and necrotic cells that die prematurely. HMGB1 signals “danger” in a paracrine manner to neighboring cells, which induces inflammation by interacting with multiple receptors. The first receptor identified for HMGB1 is the receptor for advanced glycation end products (RAGE), which sits within the immunoglobulin superfamily. HMGB1 also binds with multiple members of the TLR family. Indeed, HMGB1 can complex with CpG-DNA and bind to TLR9 and RAGE to increases cytokine production in plasmacytoid dendritic cells [132]. When HMGB1 is bound to nucleosomes, macrophages and dendritic cells are activated through TLR2 [133].

Yet, most studies focus on examining the HMGB1/TLR4 axis [134,135,136,137]. Signaling through HMGB1/TLR4 has been shown to lead to inflammation and immune regulation, including such processes as macrophage cytokine production [138], neutrophil production of reactive oxygen species [137] and NET formation [139], amongst many others. More specifically, platelet-derived HMGB1 has been implicated in promoting monocyte migration, via ligation to RAGE, and concomitantly suppressing monocyte apoptosis, via ligation to TLR4 [140]. Also, platelet production of this DAMP encourages monocyte accumulation at the site of vascular thrombosis via both RAGE and TLR2 [141] and can promote neutrophil-mediated host defense mechanisms during sepsis [142]. Platelet aggregation can also be mediated by the HMBG1/TLR4 axis [140]. HMGB1 is a regulatory trigger of the NLRP3 inflammasome. Recently, Vogel et al. [143] demonstrate that the platelet NLRP3 inflammasome is upregulated in sickle cell disease (SCD) via the HMGB1/TLR4 axis.

### 5.3. Platelet DAMP Production during Storage of Platelet Transfusion Products

Platelet concentrates account for just 10% of all blood transfusion components but are responsible for more than 25% of the reported clinical serious adverse events, which include febrile non-hemolytic transfusion reaction or allergic reactions (amongst others). Even though platelet-rich transfusion products are sterile, platelets continuously release immune-modulating components during processing and storage. For example, the levels of sCD40L that are secreted during storage can be compared to those observed in chronic inflammation (3000–7000 ng/L) [144] and these levels are sufficient to provoke immune activation of B cells, T cells and monocytes [145]. Consequently, some adverse events present with acute inflammatory symptoms, which can be severe [146,147,148].

Biological response modifiers that are secreted by platelets during storage of these platelet products has been associated with transfusion-induced adverse events (AEs). For example, levels of mtDNA in platelet components (PC) were significantly associated with the incidence of AEs [17,18]. Furthermore, Marcoux et al. [149] indicated that subtypes of platelet-derived extracellular vesicles in PC, such as those conveying mitochondria, can be a valuable biomarker for assessing the likelihood of adverse transfusion reactions. Although not demonstrated in platelets to date, there is evidence to suggest that mtDNA release is an active, rather than passive, stress response. For example, macrophages are induced to synthesize mtDNA upon stimulation with LPS [150]. To support this model, platelets are known to shed microparticles which contain mitochondria upon activation [17]. Similarly to the phenomenon described with mtDNA, Cognasse et al. [19] recently provided initial proof that soluble HMGB1 levels in PC are closely linked with the incidence of transfusion-related AEs.

Aside from their production of mtDNA, platelets may also respond to mtDNA via their expression of TLR9 [71,77,95,124] as these cells are known to both sequester CpG dinucleotides [124] and activate in response to mtDNA [151]. Similarly, HMGB1 can activate platelets via expression of TLR4 [140,143] and, via these mechanisms, platelets may actively participate as cell sensors of both mtDNA and HMGB1. This may provide a closed feedback loop within stored PCs where platelet-mediated inflammation can proceed, relatively unchecked. To consider data that may support this theory, the concentration of platelet-derived EVs containing mitochondria has been shown to positively strongly correlated with the concentration of sCD62P, a platelet activation marker, in PC [149]. However, in the same study, the same strength of correlation was not observed with sCD40L [149]. An interesting and outstanding avenue of research in this field is directly determining the extent to which platelet-TLR pathways are involved in both the secretion of, and response to, HMGB1 and mtDNA in stored PC.

## 6. TLR Therapeutics: Potential for Targeting Platelet-TLRs?

Given their active involvement in promoting inflammation in various diseases, there is considerable interest in therapeutically targeting TLRs. Several classes of TLR inhibitors are being investigated in both pre-clinical and clinical contexts [152]. TLR therapeutics that have transitioned from the bench into clinical trials have, in the main, focused on prevention and relief of infection, sepsis, autoimmunity and CVD although, so far, with mixed success. In these clinical trials, the aim of administering these TLR therapeutics is to globally dampen TLR signaling within both immune and non-immune cells expressing TLRs. We direct the reader to the following reviews [152,153,154,155] that provide a more globalized and in-depth view on TLR therapeutics. Although there has been no focus on how these therapeutics affect platelet-TLR signaling specifically, here we discuss some examples that hold promise for potentially dampening platelet responses in this context. 

One such example is eritoran, a synthetic analogue of the lipid A portion of LPS that acts to competitively bind and antagonize TLR4 [156,157]. Eritoran has been extensively investigated as a therapy for rampant endotoxin-mediated inflammation. Most notably, eritoran was tested in a phase III clinical trial with a primary endpoint of reducing 28-day all-cause mortality in patients with severe sepsis [158]. Although eritoran did not show any survival benefit in this study [158], this result has not dampened the pursual of this drug in other endotoxin-related diseases. Eritoran can reduce the severity of infection-related cytokine storms in animal models [159,160] and, as such, has been touted as therapy to be considered for treatment of COVID-19 [161]. In the context of platelet-TLR4 signaling, Clark et al. [43] demonstrated a role for eritoran in reducing platelet-mediated NET formation. Specifically, eritoran prevented LPS-mediated platelet-neutrophil aggregation and platelet-mediated neutrophil degranulation [43]. Aside from globally dampening excessive cytokine production, eritoran may also influence platelet-leukocyte interactions to support an anti-inflammatory environment.

A major focus for TLR therapeutics has been harnessing the off-target TLR-dampening effects of currently available drugs, such as angiotensin II receptor blockers (ARBs). For example, losartan can inhibit leukocytic inflammatory responses [162] via inhibition of NF-ĸB [163]. Separately, losartan is known to reduce platelet aggregability [164] via both thromboxane A2-dependent [165,166] and GPVI-dependent [167,168] pathways. Although not yet investigated in this context, TLR-mediated platelet activation may also be regulated by losartan. Losartan may act as an effective anti-platelet and anti-inflammatory treatment alongside its role as an ARB.

Aside from re-purposing currently available drugs, new therapeutics, in the form of non-coding RNAs, are promising in pre-clinical experiments. Non-coding RNAs, including microRNAs (miRs), have emerged as important post-transcriptional regulators of TLR signaling [169,170,171]. In particular, miR-223 has been implicated in negatively regulating TLR-NFκB signaling [172]. As a potential therapeutic option, miR-223 overexpression markedly reduces pro-inflammatory cytokine production in TLR-stimulated macrophages [173,174] and limits neutrophilic inflammation [175]. Aside from regulating TLRs, miR-223 is the most abundant miRNA in platelets [176,177] and is known to regulate platelet expression of the P2Y_12_ receptor [177]. Platelets from miR-223-deficient mice undergo accelerated aggregation and enhanced platelet-neutrophil aggregation in vitro [178]. In human studies, high platelet reactivity has been associated with lower plasma levels of miR-223 in healthy subjects [179] and in patients with both stable [180] and acute [179,181,182] CVD. miR-223 has not yet been linked to modulating platelet-TLR signaling, but it may be that miR-223 is involved in platelet function beyond regulating P2Y_12_ expression.

This discussion serves to highlight that, while platelet-TLRs evidently have an important role in thrombo-inflammation, there is a need to interrogate the effect of TLR therapeutics at the level of the platelet in both pre-clinical and clinical experiments.

## 7. Conclusions

TLRs have an established role in eliciting platelet thrombo-inflammatory responses and it is the current working hypothesis that platelet-TLRs participate more strongly in immunity over thrombosis. Via these pathways, platelets act as bloodstream sentinels to sense and act against exogenous and endogenous danger. In this review, we provide a comprehensive update on platelet-TLR expression, TLR-mediated platelet activation and the release of TLR-triggering DAMPs by platelets (aided by Tables S2–S6). Platelet responses are well-characterized for a subset of platelet-TLRs, while others of this receptor family remain understudied in platelets. We highlight these gaps as a series of unanswered research questions that, if pursued, can constructively expand the knowledge base of this field (Figure 3). We also provide a set of methodological considerations (Table S1) and have compiled a list of techniques used to assess platelet-TLR engagement (Tables S7 and S8), which we believe can serve as an effective resource—a “researcher’s toolkit”—for undertaking future research in this field.

Platelet-TLRs are pathomechanistic in both infection and sterile inflammation: we discuss, here, their role in influenza, sepsis, transfusion-related injury and cardiovascular disease, and demonstrate these pathways to be active and intact in a range of clinical conditions. If we extrapolate from these contexts, TLRs are potential active participants in various human diseases where platelet hyperreactivity underlies the development and pathophysiology.

To summarize, platelet-TLRs are at the nexus of vascular immunity and thrombosis and are important, potentially under-appreciated, pathways in many thrombo-inflammatory conditions.

## Supplementary Materials

Supplementary materials can be found at https://www.mdpi.com/1422-0067/21/17/6150/s1.

## Abbreviations

ACS	Acute coronary syndrome
ADP	Adenosine diphosphate
AEs	Adverse events
AF	Atrial fibrillation
ATP	Adenosine triphosphate
C3	Complement 3
CAPs	Carboxy(alkylpyrrole) protein adducts
CD62P	P-selectin
cGMP	Cyclic guanosine monophosphate
CVD	Cardiovascular disease
DAMP	Damage-associated molecular pattern
DAPT	Dual anti-platelet therapy
FSL-1	Fibroblast stimulating lipopeptide-1
GP	Glycoprotein
HCMV	Human cytomegalovirus
HMGB1	High Mobility Group Box 1
IL	Interleukin
LPS	Lipopolysaccharide
MALP-2	Macrophage Activating Lipopeptide-2
MIP	Macrophage inflammatory protein
MPO	Myeloperoxidase
MPs	Microparticles
mtDNA	Mitochondrial DNA
NET	Neutrophil extracellular trap
NF-κB	Nuclear factor-kappa B
oxPC	Oxidized phospholipids
PAMP	Pathogen-associated molecular pattern
PBMCs	Peripheral blood mononuclear cells
PC	Platelet components
PF4	Platelet factor 4
PGSL-1	P-selectin glycoprotein ligand-1
PRP	Platelet-rich plasma
PRR	Pattern recognition receptor
RAGE	Receptor for advanced glycation end products
RANTES	Regulated upon Activation, Normal T cell Expressed and Secreted
sCD40L	Soluble CD40L
TGF-β	Transforming growth factor-β
TLRs	Toll-like receptors
TNF-α	Tumor necrosis factor-alpha
TRAP	Thrombin receptor activator peptide
T_reg_	Regulatory T cells
TxA_2_	Thromboxane A_2_
VAMP	Vesicle-associated membrane protein
vWF	Von Willebrand factor
WPs	Washed platelets
