# Supplementary material for: Revisiting Platelets and Toll-Like Receptors (TLRs): At the Interface of Vascular Immunity and Thrombosis
Source: Int J Mol Sci. 2020 Aug 26;21(17):6150. doi: 10.3390/ijms21176150 (PMC7504402; doi:10.3390/ijms21176150)
Supplement: Supplementary file 1 [file ijms-21-06150-s001.pdf]

Revisiting platelets and Toll-like receptors (TLRs): at the interface of vascular immunity and thrombosis

Kathryn Hally [1, 2, 3] *, Sebastien Fauteux-Daniel [4, 5], Hind Hamzeh-Cognasse [5], Peter Larsen [1, 2, #], Fabrice Cognasse [4, 5, #].

[1] Department of Surgery and Anaesthesia, The University of Otago, Wellington, New Zealand; kathryn.hally@otago.ac.nz (K.H.); peter.larsen@otago.ac.nz (P.L.)

[2] Wellington Cardiovascular Research Group, Wellington, New Zealand

[3] School of Biological Sciences, Victoria University of Wellington, Wellington, New Zealand

[4] Établissement Français du Sang Auvergne-Rhône-Alpes, Saint-Étienne, France;

[5] Université de Lyon, GIMAP-EA3064, Saint-Etienne, France.

* Correspondence: kathryn.hally@otago.ac.nz; Tel.: +64-4-918-5610.

[#] Joint senior authors.

Supplementary Materials.

A researcher's toolkit for examining platelet-TLR expression and activation in future studies.

We have endeavoured to provide a set of tables, which have been referenced in this review, to inform researchers of the currently available information on platelet-TLRs. This list of tables aims to advise researchers on methodological considerations for study design, provide updated information on the platelet-TLR literature and provide a list of techniques that have been used to assess platelet responses to TLR agonism in previous studies. The list is as follows:

**Table S1**	Methodological considerations for examining TLR-mediated platelet activation in future studies.
**Table S2**	Platelet expression of traditional cell-surface TLRs, stratified by the technique for detection.
**Table S3**	Platelet responses to prototypical TLR2 agonism.
**Table S4**	Platelet expression of traditional intracellular TLRs, stratified by technique for detection.
**Table S5**	Platelet responses to prototypical TLR3, TLR7 and TLR9 agonism.
**Table S6**	Platelet responses to prototypical TLR4 agonism.
**Table S7**	A description of the ways in which direct platelet responses to TLR agonism have been examined previously.
**Table S8**	A description of the ways in which the effect of platelets on neutrophils and PBMCs in response to TLR agonism has been examined previously.

Table S1. Methodological considerations for examining TLR-mediated platelet activation in future studies.

	Aspect of methodology	Considerations	Recommendation
1	TLR blockade	• Pre-incubation with a monoclonal antibody is commonly used to assess the specificity of platelet responses to TLR agonism.<br>• This has most often been assessed for TLRs 2 and 4, and common clones are:<br>o TLR2: T2.5 [1-6] and TL2.1 [7-9].<br>o TLR4: HTA125 [1,2,4,5,9,10] and 18H10 [3,6].<br>• TLR blockade has mostly been used to demonstrate the contribution of these platelet-TLR pathways in response to bacterial/viral stimulation [2,3,9,11-13] but has also been used to demonstrate the specificity of prototypical TLR2/1 [1,4,7,8] and TLR4 [1,4-6,14,15] agonists.<br>• TLR blockade has also been used to demonstrate the specificity of pathogenic ligands, such as $oxPC_{CD36}$ signalling via TLR2/6 [16] and CAP signalling via TLR9 [17].	**Monoclonal antibody blockade should be used to demonstrate the specificity of the platelet responses to TLR agonism.**
2	Type of platelet preparation used (WPs, PRP and WB)	• TLR stimulation is widely studied with WPs and PRP, and less so with WB.<br>• Where WPs are used, the preparation protocols vary hugely.<br>o Particularly with respect to the type of anticoagulant, platelet inhibitor and resuspension buffer used.<br>• Many studies have compared responses in WPs and PRP with contrasting results. Examples of these studies are:<br>o In one study, the responses to LPS in PRP was abolished in WPs, but could be restored with the addition of CD14 to WPs [18]. In another study, WPs were supplemented with CD14 and LBP to examine the effects of LPS [19].<br>o In contrast, effects of Pam3CSK4 and LPS were elevated in WPs compared to PRP [20].<br>• TLR-mediated platelet activation is seen in PRP and is potentiated in WB [21], suggesting an indirect effect of TLR stimulation in the presence of other TLR-expressing cells.	**The different responses seen within studies suggests the need to examine both WPs and PRP to demonstrate the contribution of plasma factors and the effect of manipulating platelets to produce WPs.**
3	Type of TLR agonist	• The choice of agonist varies with the type of TLR under examination. For most TLRs, a range of prototypical agonists exist.<br>o TLR2/6 agonists include FSL-1 [22,23], MALP-2 [24], Pam2CSK4 [7,16] and LTA [3].<br>o TLR3 agonists include Poly(I:C) [25,26] and Poly(A:U) [26].<br>o TLR7 agonists include loxiribine [27,28] and R837 (imiquimod) [28].<br>• Platelet responses to TLR2/1 engagement are exclusively assessed with Pam3CSK4 stimulation.<br>• For assessing platelet-TLR4 responses, LPS is also exclusively used but the type of LPS varies between studies. Some examples are:<br>o LPS isolated from the *E. coli* O111:B4 strain is often used [1,4,5,14,15,18,20,29,30] and is widely commercially available.<br>o Several LPS types were tested by Stahl et al. [31]: in order of potency (PAC1 binding and CD40L expression) O57:H7 > O121:H19 > O111:H7 > O103:H2.<br>o Rough vs. smooth LPS have different potencies [32]: Rough, but not smooth, LPS can potentiate platelet aggregation and induce expression of CD40L and release of microparticles.<br>o Other LPS types tested include from *Escherichia coli* K12 [30] and R515 [21-23,33], *Salmonella minnesota* R595 [34], *Klebsiella pneumoniae* [6], and *Pseudomonas aeruginosa* [6].<br>o Using the bacterial strain *E. coli* O111 (a clinical isolate) was shown to induce platelet pro-thrombotic responses in a way that commercially-available LPS was not able to do [10].	**For most TLRs, use of more than one prototypical agonist (or in the case of LPS, more than one source).**

4	Concentration of agonist	• It is often difficult to ascertain the physiological level of circulating TLR agonists in various clinical conditions. This is compounded by the notion that, for some clinical conditions, TLR ligands are likely to accumulate at the site of infection or injury (for example, within atherosclerotic lesions) so that platelets may be exposed to high localized doses.<br>• Stimulation with Pam3CSK4 is usually tested at doses up to 10 µg/mL [1,3,4,7,24,27-29,35-39] and some tested up to 100 µg/mL [8,20,21,33].<br>• For LPS, similar doses are tested:<br>o Between 1 and 10 µg/mL [1,3,4,6,14,18,29-32,34].<br>o Up to 50 [10] and 100 µg/mL [5,20,21,33].<br>o Hally et al. [21] show platelet activation in response to 100 µg/mL LPS, which was not seen at any other dose tested (0.1 to 50 µg/mL).<br>• Some studies have tested the effect of LPS at much lower doses:<br>o Nocella et al. [15] demonstrate that plasma and serum LPS levels are 107 and 145 pg/mL, respectively, in patients with pneumonia. Thus, in this study, platelet activation was examined in response to up to 100 pg/mL LPS [15]. Potentiation of platelet responses could be observed at a dose of 15 pg/mL over a short stimulation time.<br>o Overnight exposure of platelets with 100 ng/mL LPS increased microparticle shedding and release of IL-1β [19].<br>• The effect of platelets on PBMC and neutrophil responses to Pam3CSK4 and LPS are often assessed over longer periods of time (4 to 24 hours) with concentrations between 1 and 100 ng/mL [22,23,38].	**For TLR2/1 and TLR4, examine dose-dependent response with concentrations of ≤10 µg/mL.**<br><br>**Examining the platelet effect in co-culture models is likely to require much lower (ng/mL) concentrations and longer stimulation times.**
5	Outcome measurements	• TLR engagement induces numerous thrombotic and immune functions, which are listed in Table S 1.<br>• The most widely assessed thrombotic functions are:<br>o Platelet aggregation and ATP/ADP release (lumi-aggregometry).<br>o Fibrinogen or PAC1 binding (flow cytometry).<br>o Platelet-surface P-selectin and CD63 exposure (as markers of alpha and dense granule secretion) (flow cytometry).<br>• The most widely assessed immune functions are:<br>o Platelet-leukocyte aggregation (flow cytometry).<br>o Platelet-surface P-selectin and CD40L exposure (as markers of ability to ligate with leukocytes) (flow cytometry).<br>o Release of inflammatory mediators such as RANTES, PF4, sCD40L and IL-1β (ELISA).	**The use of a combination of measurements (including thrombotic and immune functions) allows for a global assessment of platelet-TLR engagement.**
6	Stimulation time	• Most functions are measured over short stimulation times. For example:<br>o Platelet aggregation and ATP/ADP release (≤15 minutes).<br>o P-selectin and CD40L exposure (≤ 20 minutes).<br>• However, many immune functions are measured over longer stimulation times:<br>o RANTES release (2 to 12 hours [8,37,40]).<br>o Cytokine release by PBMCs in platelet co-culture (24 hours [23,38]).<br>o Neutrophil activation in platelet co-culture (4 hours [22,23]).<br>o sCD40L release (1 to 3 hours [8,11]).<br>o IL-1β release (3 to 18 hours [19,25]).	**It is necessary to optimize some types of immune functions over longer stimulation periods than is traditionally assessed in platelet research.**

$oxPC_{36}$, oxidised phospholipids activated by CD36; CAP, carboxy(alkylpyrole) protein; WPs, washed platelets; PRP, platelet-rich plasma; WB, whole blood; LPS, lipopolysaccharide; LBP, lipopolysaccharide binding protein; FSL-1, fibroblast-stimulating lipopeptide-1; MALP-2, macrophage activating lipopeptide-2; LTA, lipoteichoic acid; CD40L, CD40 ligand; IL-1β, interleukin-1 beta; PBMC, peripheral blood mononuclear cell; ATP; adenosine triphosphate; ADP, adenosine diphosphate; RANTES, Regulated upon Activation, Normal T cell Expressed and presumably Secreted; PF4, platelet factor 4; sCD40L, soluble CD40L.

Table S2. Platelet expression of traditional cell-surface TLRs, stratified by technique for detection.

TLR	Flow cytometry	Fluorescence microscopy	Western blot	PCR
1	• No [29] or very low cell-surface [7,41] expression<br>• Very low intracellular expression [41]	• Expressed on platelets found in the coronary thrombi of ACS patients [42]	• 53% increase in AMI vs. control platelets [21]<br>• Presence confirmed [17,42]	• Presence in ~50% of participants in a Framingham Heart sub-study [43]<br>• Presence confirmed [42]
2	• Moderate cell-surface expression [7,16,31,41,44] vs. no expression [29]<br>• High intracellular expression [41]<br>• Elevated [6,20,41] vs. not elevated [44] in activated platelets<br>• ↑ in ACS vs. stable angina and control platelets [45]<br>• No change in patients with Essential Thrombocythemia [46]<br>• Cell-surface expression higher on WPs vs. PRP [20]		• Presence confirmed [17,21]<br>• No difference in expression in AMI vs. control platelets [21]	• Presence in ~70% of participants in a Framingham Heart sub-study [43] Significant elevation in ACS vs. control platelets [47]
4	• Significant cell-surface expression [3,20,31,41,44,48] vs. no expression [29]<br>• Significant intracellular expression [41] Modulation of expression in activated platelets [31,41,44]<br>• Cell-surface expression higher on WPs vs. PRP [20]<br>• Cell-surface expression investigated in sepsis [6,49,50], CVD [45], Crohn's disease [51] and Essential Thrombocythemia [46]		• Significant increase in CAP vs. control [15] and in AMI vs. control [21] platelets<br>• Presence confirmed [5,44]	• Presence in ~60% of participants in a Framingham Heart sub-study [43]<br>• Presence confirmed [47]
5	• Low cell-surface expression, elevated in activated platelets [6]			• Presence in ~85% of participants in a Framingham Heart sub-study [43]
6	• Very low cell-surface [7,16,41] and intracellular [41] expression	• Expressed on platelets found in coronary thrombi of ACS patients [42]	• Presence confirmed [17,21,42] No difference in expression in AMI vs. control platelets [21]	• Presence of TLR5 mRNA in ~20% of participants within a Framingham Heart sub-study [43]<br>• Presence confirmed [42]
10				• Presence in ~80% of participants in a Framingham Heart sub-study [43]

For the first column, **navy blue shading**: high expression; **dark blue shading**: moderate expression; **light blue shading**: low expression; **white shading**: extent of expression relatively unconfirmed. For all other columns, green shading: expression confirmed by this technique. ACS: acute coronary syndromes; AMI: acute myocardial infarction; WPs: washed platelets; PRP: platelet-rich plasma; CAP: community-acquired pneumonia.

Table S3. Platelet responses to prototypical TLR2 agonism.

TLR	(Lumi) Aggregometry	Spectrofluorometry	Flow cytometry	ELISA of releasate	Other techniques
**2/1**	• ↑ Aggregation [1,2,7,20,24,35-37] vs. no aggregation [3,29] (in PRP [20])<br>• ↑ ATP [1,24,36] and ADP [2] release	• ↑ Intracellular [Ca^{2+}] mobilization [24,36,37] vs. no mobilization [29]	• ↑ P-selectin expression [1,3,7,8,21,33,46] vs. no expression [29]<br>• ↑ CD40L expression [1,46,52]<br>• ↑ CD63 expression [46]<br>• ↑ Fibrinogen [1] and PAC1 [21,33,46] binding<br>• ↑ Platelet-neutrophil [1,12,28,46] and platelet-monocyte [21,28,35,38] aggregation<br>• ↑ CD63 [12] vs. no CD63 expression [3]<br>• ↑ Monocyte [38] and neutrophil [22] phagocytosis<br>• ↓ Neutrophil activation [22,23]	• ↑ TxB2 [24]<br>• ↑ sCD40L [8,52] (only in WPs [20])<br>• ↑ vWF [1]<br>• ↑ RANTES [8,20,37,40,46]<br>• ↑ MIF [37,40]<br>• ↑ PAI-1 [37]<br>• ↑ PF4 [20] vs. no release [8]<br>• ↑ PDGF [20]<br>• ↑ NAP-2 (only in WPs [20])<br>• ↓ IL-1β, TNFα, IL-6, IL-10 and IL-1Ra by PBMCs [38]<br>• ↓ Neutrophil elastase [22,23]<br>• ↑ Neutrophil MPO [27]<br>• ↑ NF-κB p65 phosphorylation [8]	• ↑ Neutrophil DNA release [4]<br>• ↑ Platelet adhesion to collagen-coated slides [35]
**2/6**	• No platelet aggregation [3,24] vs. aggregation (but not as effective/consistent as TLR2/1 engagement) [7]<br>• No ATP release [24]		• No P-selectin expression [3,21] vs. expression [16] only at high-dose in whole blood [21]<br>• PAC1 binding only at high-dose in whole blood [21]<br>• No CD63 expression [3]<br>• No heterotypic aggregation [3] vs. heterotypic aggregation at high-dose [21]<br>• ↓ Neutrophil activation [22,23] vs. ↑ Neutrophil phagocytosis [22]	• ↓ TNFα vs. ↑ IL-10, MIP-1β, IL-6 release by PBMCs [23]<br>• ↓ Neutrophil elastase [22,23]	

Green shading: activation investigated by this technique. ATP, adenosine triphosphate; ADP, adenosine diphosphate; CD40L, CD40 ligand; TxB2, thromboxane B2; sCD40L, soluble CD40L; vWF: von Willebrand factor; RANTES, Regulated upon Activation, Normal T cell Expressed and presumably Secreted; MIF, macrophage migration inhibitory factor; PAI-1, plasminogen activator inhibitor-1; PF4, platelet factor 4; PDGF, platelet-derived growth factor; NAP-2, neutrophil activating peptide-2; IL-1β, interleukin-1 beta; TNFα, tumour necrosis factor alpha; IL-6, interleukin-6; IL-10, interleukin-10; IL-1Ra, interleukin-1 receptor antagonist; PBMCs, peripheral blood mononuclear cells; MPO, myeloperoxidase; NF-κB, nuclear factor-kappa B; MIP-1β, macrophage inflammatory protein-1 beta.

Table S4. Platelet expression of traditional intracellular TLRs, stratified by technique for detection.

TLR	Flow cytometry	Fluorescence microscopy	Western blot	PCR
**3**	• Very low cell-surface and moderate intracellular expression [25,26]<br>• Elevated in activated platelets [25]	• Presence confirmed [26]		• Presence in ~50% of participants in a Framingham Heart sub-study [43]<br>• Presence confirmed [25]
**7**			• Presence confirmed [17]	• Variable expression of TLR7 mRNA (25% [43] vs. 60% [28] of cohort) in sub-studies of the Framingham Heart Study
**8**	• Very low cell-surface and intracellular expression [41]		• Dose-dependent increase in response to pre-treatment with chitin from *Candida albicans* and stimulated with thrombin [53]	• Presence in ~15% of participants in a Framingham Heart sub-study [43]<br>• Presence confirmed [53]
**9**	• Low/moderate cell-surface expression [6,17,41,44,54]<br>• Moderate intracellular expression [41,55]<br>• Elevated cell-surface [6,17,41,44,54] and intracellular [41,55] expression in activated platelets	• Punctuate/granular intracellular expression [17,54], congregating adjacent to the plasma membrane [54]	• Presence confirmed [17,55]	• Presence in ~90% of participants in a Framingham Heart sub-study [43]<br>• Presence confirmed [17,47]

For the first column, **blue shading**: low/moderate expression; **light blue**: low expression; **white shading**: extent of expression relatively unconfirmed. For all other columns, green shading: expression confirmed by this technique.

Table S5. Platelet responses to prototypical TLR3, TLR7 and TLR9 agonism.

TLR	(Lumi) Aggregometry	Spectrofluorometry	Flow cytometry	ELISA of releasate	Other techniques
**3**	• Potentiated platelet aggregation and ATP release [26]	• Intracellular [Ca^{2+}] mobilization [25]	• ↑ P-selectin expression [25] vs. no expression [26]<br>• Potentiated fibrinogen binding [26]<br>• No CD40L expression [26]	• ↑ PF4 [25]<br>• ↑ IL-1β [25]	
**7**	• No platelet aggregation + no potentiation [28]		• ↑ Platelet-leukocyte aggregation [28]<br>• ↑ P-selectin expression [28]<br>• ↑ CD40L expression [28]<br>• No PAC1 binding [28]	• ↑ Complement 3 release in platelets expressing TLR7 mRNA [27]<br>• ↑ Neutrophil GM-CSF and MPO release [27]	• Platelet binding to collagen-coated slide [28]<br>• ↑ Neutrophil DNA release [27]
**9**	• Potentiation of platelet aggregation [17]		• ↑ P-selectin expression [17,54,55]<br>• ↑ CD63 expression [55]<br>• ↑ CpG sequestration [54]		

Green shading: activation investigated by this technique. ATP, adenosine triphosphate; CD40L, CD40 ligand; PF4, platelet factor 4; IL-1β, interleukin-1 beta; GM-CSF; granulocyte-macrophage colony-stimulating factor; MPO, myeloperoxidase.

Table S6. Platelet responses to prototypical TLR4 agonism.

TLR	(Lumi) Aggregometry	Spectrofluorometry	Flow cytometry	ELISA of releasate	Other techniques
4	• No platelet aggregation [1,3,5,6,14,15,20,29,32]<br>• Platelet aggregation in WPs (but not PRP) [20]<br>• Potentiation [1,5,15,30,32] vs. no potentiation of platelet aggregation [3,6,29]<br>• ATP release [5] + potentiation [1]	• No [Ca^{2+}] mobilization [29]	• ↑ PAC1 expression [31] (only at high-dose) [21,33]<br>• ↑ CD40L expression [31,32,52]<br>• ↑ Fibrinogen binding [30,31] + potentiation [1,30] vs. no binding [34]<br>• ↑ P-selectin expression [10] (only at high-dose) [21,33] + potentiation [30] vs. no expression [3,6,10,14,29,32,34]<br>• ↑ Heterotypic [12,14,46] vs. no heterotypic [3,6,21,38] aggregation<br>• ↑ Microparticle shedding [19,32] + potentiation [32]<br>• ↑ CD63 expression [18,56] vs. no expression [3,6]<br>• Potentiation of ROS production [30]<br>• No monocyte phagocytosis [38]<br>• ↓ Monocyte and neutrophil activation [23]	• ↑ cGMP production [5]<br>• ↑ IL-1β [19]<br>• ↑ sCD40L [18,52,56] (only in WPs [20])<br>• ↑ vWF release [1]<br>• ↑ RANTES [20,46,56]<br>• ↑ PDGF [20]<br>• ↑ PF4 [20] vs. no release [56]<br>• ↑ NAP-2 (only in WPs) [20]<br>• Potentiated TxB2 [15]<br>• Potentiated 8-iso-PGF2α [15]<br>• Potentiated H_2O_2 [15]<br>• Potentiated sNOX2-dp [15]<br>• ↑ IL-1β and IL-6 vs. ↓ IL-10 by PBMCs [38]<br>• ↓ IL-6, TNFα and MIP-1β vs. ↑ IL-10 by PBMCs [23]	• ↑ Neutrophil degranulation, MMP-9 release, bacterial trapping and DNA release by neutrophils [14]<br>• Shortening clotting parameters [10]<br>• ↑ FXa generation [10]<br>• ↑ Maximal oxygen consumption rate [6]

Green shading: activation investigated by this technique. WPs, washed platelets; PRP, platelet-rich plasma; ATP, adenosine triphosphate; CD40L, CD40 ligand; ROS, reactive oxygen species; cGMP, cyclic guanosine monophosphate; IL-1β, interleukin-1 beta; sCD40L, soluble CD40L; vWF, von Willebrand factor; RANTES, Regulated upon Activation, Normal T cell Expressed and presumably Secreted; PDGF, platelet-derived growth factor; PF4, platelet factor 4; NAP-2, neutrophil activating peptide-2; TXB2, thromboxane B2; 8-iso-PGF2α, 8-iso-prostaglandin 2 alpha; H_2O_2, hydrogen peroxide; sNOX2-dp, soluble NOX2-derived peptide; IL-1β, interleukin-1 beta; IL-6, interleukin-6; IL-10, interleukin-10; PBMCs, peripheral blood mononuclear cells; TNFα, tumour necrosis factor alpha; MIP-1β, macrophage inflammatory protein-1 beta; MMP-9, matrix metalloproteinase 9; FXa, factor Xa.

Table S7. A description of the ways in which direct platelet responses to TLR agonism have been examined previously.

Technique	Measurement*	Specifics	References
(Lumi) Aggregometry	Platelet aggregation	Turbidometric platelet aggregometry detects the difference in light transmission after the addition of a platelet agonist to PRP.	[1-3,5-7,14,15,17,20,24,26,28-30,32,36,37,57]
(Lumi) Aggregometry	Adenosine triphosphate (ATP) release	ATP is commonly quantified by using a luciferin/luciferase assay, where luciferin and released ATP are substrates for luciferase, which catalyses light formation.	[1,2,5,24,26,36]
Flow cytometry and spectrofluorometry	Intracellular [Ca^{2+}] mobilization	Platelets are labelled with calcium indicators that exhibit an increase in fluorescence upon binding to Ca^{2+}. Platelets are loaded with the cell-permeant dyes, fluo-3 acetoxymethyl (AM) ester (fluo-3 AM) or fura-2 AM.	[24,25,29,36,37]
Flow cytometry	Platelet-surface P-selectin (CD62P)	A protein specific to platelet α-granules that is translocated to the platelet surface upon activation.	[1,3,6,7,10,14,16,17,21,26,28-30,32-34,46,54,55]
Flow cytometry	Platelet-neutrophil and platelet-monocyte aggregation	Platelet-specific marker (e.g. CD41, CD42a, CD42b) paired with various leukocyte-specific markers (e.g. CD45, CD14, CD64, CD16) in either whole blood or washed preparations.	[1,3,6,7,14,21,28,38,46,57]
Flow cytometry	Platelet-surface CD40 ligand (CD40L; CD154)	CD40L is cryptic in unstimulated platelets but is rapidly mobilized to the platelet surface upon activation.	[1,26,28,31,32,46,52]
Flow cytometry	Fibrinogen binding	Platelets are incubated in the presence of fluorescently labelled fibrinogen.	[1,26,30,31,34]
Flow cytometry	PAC1 binding	PAC1 is a protein that identifies the activation-dependent epitome GPIIb/IIIa on the surface of activated platelets.	[21,28,31,33,46]
Flow cytometry	Platelet-surface CD63	A protein specific to platelet dense granules that is translocated to the platelet surface upon activation.	[3,6,8,46,55]
Flow cytometry	Platelet microparticle shedding	Microparticles in platelet releasate were counted using a known amount of polystyrene latex beads.	[19,32]
Flow cytometry	Phosphatidylserine (PS) exposure	PS exposure on the platelet surface indicates increased coagulability.	[6]
Flow cytometry	Total reactive oxygen species (ROS) production	Platelets incubated with DCFH-DA, a cell-permeable non-fluorescent probe that becomes highly fluorescent upon oxidation.	[30]
Flow cytometry	Association of platelets with platelet microparticles	Platelets were labelled with cell-permeant, green-fluorescent DIOC6 and microparticles were labelled with DiD red-fluorescent cell-labelling solution. Association was measured as number of double-positive events.	[57]
Measurements in the platelet releasate (detection by ELISA)	RANTES (CCL5)	Found within the α-granules of platelets, released upon activation and promotes leukocyte recruitment.	[8,20,37,40,46]
Measurements in the platelet releasate (detection by ELISA)	Platelet Factor 4 (PF4; CXCL4)	PF4 is a chemokine released from platelet α-granules and promotes blood coagulation.	[8,20,25]
Measurements in the platelet releasate (detection by ELISA)	Soluble CD40L (sCD40L)	CD40L is cleaved from the platelet surface into sCD40L, which has cytokine-like activity.	[8,20,52]

Measurements in the platelet releasate (detection by ELISA)	Thromboxane B2 (TxB2)	TxB2 is the stable, inactive metabolite of TxA2. TxA2 is involved in potentiating platelet activation and is a product of arachidonic acid oxidation by COX-1.	[15,24]
	Interleukin 1β (IL-1β)	Platelets can splice IL-1β mRNA to produce newly synthesized mature IL-1β protein upon stimulation.	[19,25]
	Macrophage migration inhibitory factor (MIF)	MIF was shown to be secreted by platelets in response to Pam3CSK4, detectable in a preformed 36 cytokine array. Platelet-derived MIF has chemotactic and clotting properties.	[37,40]
	Soluble NOX-2 derived peptide (sNOX2-dp)	sNOX2-dp is a product of NOX2-generated oxidative stress.	[15]
	Hydrogen peroxide (H_2O_2)	H_2O_2 is a ROS product and is measured by colorimetric detection.	
	von Willebrand factor (vWF)	Found within the α-granules of platelets and, when released, binds to activated platelet receptors and functions in coagulation.	[1]
	Soluble P-selectin (sCD62p)	sCD62p can be released from α-granules or cleaved from cell-surface CD62p.	[8]
	Plasmingen activator inhibitor 1 (PAI-1)	PAI-1 is the key inhibitor of tissue-type plasminogen activator (tPA), a protein involved in clot fibrinolysis, and is synthesized abundantly in platelets. PAI-1 acts to stabilize platelet-rich clots against thrombolysis.	[37]
	Serotonin	Within dense granules of resting platelets, released when activated and measured by competitive ELISA.	[8]
	Platelet-derived growth factor (PDGF)	PDGF is a growth factor involved in blood vessel mitogenesis and proliferation found in platelet α-granules of resting platelets.	[20]
	Neutrophil activating peptide 2 (NAP-2; CXCL7)	NAP-2 is a proteolytically processed fragment of Platelet Basic Protein (PBP) which is found in platelet α-granules of resting platelets.	[20]
	Complement 3 (C3)	C3 plays a central role in activation of the complement system, platelet release of C3 important in enhancing NETosis.	[27]
	Isoprostane (8-iso-PGF2α-III)	This is the chemically stable product of arachidonic interaction with ROS.	[15]
Fluorescence microscopy	Adherence to collagen- and fibrinogen-coated slides	Calcein-AM labelled platelets stimulated and recirculated over coated coverslips using a cell adhesion flow chamber.	[7,28]
	Platelet-neutrophil aggregation	Calcein-AM labelled platelets were perfused over coverslip-adherent neutrophils, stationary platelets identified as platelet-neutrophil aggregation.	[14]
Extracellular flux analyser	Maximal oxygen consumption rate (OCR)	OCR is an important indicator of cell function, and highlights immediate changes in platelet function upon activation.	[6]
Thromboelastometry	Platelet-mediated clotting	Thrombin generation, FXa generation and clot formation and thickness	[10,57]

*Refer to Table S 1 for an indication of the type and concentration of TLR agonists used to assess these platelet responses.

Table S8. A description of the ways in which the effect of platelets on neutrophils and PBMCs in response to TLR agonism has been examined previously.

Technique used	Measurement*	Specifics	References
**Measurement of neutrophil functions.**			
Spectrofluorometry	Extracellular DNA in releasate	Cell-free DNA quantified using SYBR Gold	[4]
Fluorescence microscopy	Extracellular DNA of adherent neutrophils.	Cell-free DNA quantified using SYTOX Green and DAPI	[14,27]
	Neutrophil degranulation	Adherent neutrophils stained with Wright-Giemsa stain, eosin content quantified by measuring the mean fluorescent intensity of the fluorescent image	[14]
Microscopy	Bacterial trapping	Adherent neutrophils were first perfused with platelets and secondly perfused with *E. coli*. Stationary bacteria counted as bacterial trapping.	[14]
ELISA	Elastase and elastase-DNA complexes	Elastase is an enzyme that has a potent role in host defence, and is also present in abundance in neutrophil extracellular traps (NETs).	[22,23,27]
	Myeloperoxidase (MPO) level and activity	MPO is an enzyme that is present in abundance in NETs, and has a well-established role in pathogenic killing once released from neutrophils. Additionally, PMO has been linked to inflammation and increased risk in myocardial infarction.	[27]
Flow cytometry	CD66b and CD11b expression, CD62L shedding.	These are well-established markers of neutrophil activation	[22,23]
	Phagocytosis	Neutrophils + platelets incubated with IgG coated latex beads, followed by Trypan blue staining to quench cell-surface bound beads.	[22]
Zymography	Matrix metalloproteinase 9 (MMP9) release	MMP9 is involved in matrix degradation and tissue remodelling, released by neutrophils upon activation.	[14]
**Measurement of PBMC functions.**			
ELISA	Release of interleukin (IL)-6, tumour necrosis factor alpha (TNFα), macrophage inflammatory protein-1beta (MIP-1β), IL-1β, IL-10	These are common pro- and anti-inflammatory cytokines and chemokines.	[23,38]
Flow cytometry	HLA-DR expression	This is a well-established marker of monocyte activation	[23]
	Leukocyte phagocytosis of platelets	Platelets were pre-incubated with CMFDA for internal labelling prior to their addition to PBMCs. PBMCs and platelets then labelled with CD14 and CD41 to identify monocytes with internalized (CMFDA+) vs. cell-bound (CD41+) platelets.	[38]

*Refer to Table S 1 for an indication of the type and concentration of TLR agonists used to assess these platelet responses.

References

1. Rivadeneyra, L.; Carestia, A.; Etulain, J.; Pozner, R.G.; Fondevila, C.; Negrotto, S.; Schattner, M. Regulation of platelet responses triggered by Toll-like receptor 2 and 4 ligands is another non-genomic role of nuclear factor-kappaB. *Thromb Res* **2014**, *133*, 235-243, doi:10.1016/j.thromres.2013.11.028.
2. Keane, C.; Tilley, D.; Cunningham, A.; Smolenski, A.; Kadioglu, A.; Cox, D.; Jenkinson, H.F.; Kerrigan, S.W. Invasive Streptococcus pneumoniae trigger platelet activation via Toll-like receptor 2. *J Thromb Haemost* **2010**, *8*, 2757-2765, doi:10.1111/j.1538-7836.2010.04093.x.
3. de Stoppelaar, S.F.; Claushuis, T.A.; Schaap, M.C.; Hou, B.; van der Poll, T.; Nieuwland, R.; van 't Veer, C. Toll-Like Receptor Signalling Is Not Involved in Platelet Response to Streptococcus pneumoniae In Vitro or In Vivo. *PLoS One* **2016**, *11*, e0156977, doi:10.1371/journal.pone.0156977.
4. Carestia, A.; Kaufman, T.; Rivadeneyra, L.; Landoni, V.I.; Pozner, R.G.; Negrotto, S.; D'Atri, L.P.; Gomez, R.M.; Schattner, M. Mediators and molecular pathways involved in the regulation of neutrophil extracellular trap formation mediated by activated platelets. *J Leukoc Biol* **2016**, *99*, 153-162, doi:10.1189/jlb.3A0415-161R.
5. Zhang, G.; Han, J.; Welch, E.J.; Ye, R.D.; Voyno-Yasenetskaya, T.A.; Malik, A.B.; Du, X.; Li, Z. Lipopolysaccharide stimulates platelet secretion and potentiates platelet aggregation via TLR4/MyD88 and the cGMP-dependent protein kinase pathway. *J Immunol* **2009**, *182*, 7997-8004, doi:10.4049/jimmunol.0802884.
6. Claushuis, T.A.M.; Van Der Veen, A.I.P.; Horn, J.; Schultz, M.J.; Houtkooper, R.H.; Van 't Veer, C.; Van Der Poll, T. Platelet Toll-like receptor expression and activation induced by lipopolysaccharide and sepsis. *Platelets* **2019**, *30*, 296-304, doi:10.1080/09537104.2018.1445841.
7. Blair, P.; Rex, S.; Vitseva, O.; Beaulieu, L.; Tanriverdi, K.; Chakrabarti, S.; Hayashi, C.; Genco, C.A.; Iafrati, M.; Freedman, J.E. Stimulation of Toll-like receptor 2 in human platelets induces a thromboinflammatory response through activation of phosphoinositide 3-kinase. *Circ Res* **2009**, *104*, 346-354, doi:10.1161/CIRCRESAHA.108.185785.
8. Damien, P.; Cognasse, F.; Payrastre, B.; Spinelli, S.L.; Blumberg, N.; Arthaud, C.-A.; Eyraud, M.-A.; Phipps, R.P.; McNicol, A.; Pozzetto, B., et al. NF-κB Links TLR2 and PAR1 to Soluble Immunomodulator Factor Secretion in Human Platelets. *Frontiers in Immunology* **2017**, *8*, doi:10.3389/fimmu.2017.00085.
9. Liu, X.; Liu, H.; Luo, X.; Zhang, P.; Gao, Y.; Xie, S.; Xu, K.; Chang, J.; Ma, L. Strains of Group B streptococci from septic patients induce platelet activation via Toll-like Receptor 2. *Clinical and Experimental Pharmacology and Physiology* **2017**, *44*, 335-343, doi:10.1111/1440-1681.12707.
10. Matus, V.; Valenzuela, J.G.; Hidalgo, P.; Pozo, L.M.; Panes, O.; Wozniak, A.; Mezzano, D.; Pereira, J.; Saez, C.G. Human platelet interaction with E. coli O111 promotes tissue-factor-dependent procoagulant activity, involving Toll like receptor 4. *PLoS One* **2017**, *12*, e0185431, doi:10.1371/journal.pone.0185431.
11. Assinger, A.; Laky, M.; Badrnya, S.; Esfandeyari, A.; Volf, I. Periodontopathogens induce expression of CD40L on human platelets via TLR2 and TLR4. *Thromb Res* **2012**, *130*, e73-78, doi:10.1016/j.thromres.2012.04.017.
12. Assinger, A.; Laky, M.; Schabbauer, G.; Hirschl, A.M.; Buchberger, E.; Binder, B.R.; Volf, I. Efficient phagocytosis of periodontopathogens by neutrophils requires plasma factors, platelets and TLR2. *J Thromb Haemost* **2011**, *9*, 799-809, doi:10.1111/j.1538-7836.2011.04193.x.
13. Assinger, A.; Kral, J.B.; Yaiw, K.C.; Schrottmaier, W.C.; Kurzejamska, E.; Wang, Y.; Mohammad, A.A.; Religa, P.; Rahbar, A.; Schabbauer, G., et al. Human cytomegalovirus-platelet interaction triggers toll-like receptor 2-dependent proinflammatory and proangiogenic responses. *Arterioscler Thromb Vasc Biol* **2014**, *34*, 801-809, doi:10.1161/atvbaha.114.303287.
14. Clark, S.R.; Ma, A.C.; Tavener, S.A.; McDonald, B.; Goodarzi, Z.; Kelly, M.M.; Patel, K.D.; Chakrabarti, S.; McAvoy, E.; Sinclair, G.D., et al. Platelet TLR4 activates neutrophil extracellular traps to ensnare bacteria in septic blood. *Nat Med* **2007**, *13*, 463-469, doi:10.1038/nm1565.
15. Nocella, C.; Carnevale, R.; Bartimoccia, S.; Novo, M.; Cangemi, R.; Pastori, D.; Calvieri, C.; Pignatelli, P.; Violi, F. Lipopolysaccharide as trigger of platelet aggregation via eicosanoid over-production. *Thromb Haemost* **2017**, *117*, 1558-1570, doi:10.1160/th16-11-0857.
16. Biswas, S.; Zimman, A.; Gao, D.; Byzova, T.V.; Podrez, E.A. TLR2 Plays a Key Role in Platelet Hyperreactivity and Accelerated Thrombosis Associated With Hyperlipidemia. *Circ Res* **2017**, *121*, 951-962, doi:10.1161/circresaha.117.311069.
17. Panigrahi, S.; Ma, Y.; Hong, L.; Gao, D.; West, X.Z.; Salomon, R.G.; Byzova, T.V.; Podrez, E.A. Engagement of platelet toll-like receptor 9 by novel endogenous ligands promotes platelet hyperreactivity and thrombosis. *Circ Res* **2013**, *112*, 103-112, doi:10.1161/CIRCRESAHA.112.274241.
18. Damien, P.; Cognasse, F.; Eyraud, M.A.; Arthaud, C.A.; Pozzetto, B.; Garraud, O.; Hamzeh-Cognasse, H. LPS stimulation of purified human platelets is partly dependent on plasma soluble CD14 to secrete their main secreted product, soluble-CD40-Ligand. *BMC Immunol* **2015**, *16*, 3, doi:10.1186/s12865-015-0067-2.
19. Brown, G.T.; McIntyre, T.M. Lipopolysaccharide signaling without a nucleus: kinase cascades stimulate platelet shedding of proinflammatory IL-1beta-rich microparticles. *J Immunol* **2011**, *186*, 5489-5496, doi:10.4049/jimmunol.1001623.

20. Koessler, J.; Niklaus, M.; Weber, K.; Koessler, A.; Kuhn, S.; Boeck, M.; Kobsar, A. The Role of Human Platelet Preparation for Toll-Like Receptors 2 and 4 Related Platelet Responsiveness. *TH Open* **2019**, *3*, e94-e102, doi:10.1055/s-0039-1685495.
21. Hally, K.E.; La Flamme, A.C.; Larsen, P.D.; Harding, S.A. Platelet Toll-like receptor (TLR) expression and TLR-mediated platelet activation in acute myocardial infarction. *Thromb Res* **2017**, *158*, 8-15, doi:10.1016/j.thromres.2017.07.031.
22. Hally, K.E.; Bird, G.K.; La Flamme, A.C.; Harding, S.A.; Larsen, P.D. Platelets modulate multiple markers of neutrophil function in response to in vitro Toll-like receptor stimulation. *PLoS One* **2019**, *14*, e0223444, doi:10.1371/journal.pone.0223444.
23. Hally, K.E.; La Flamme, A.C.; Harding, S.A.; Larsen, P.D. Platelets regulate leucocyte responses to Toll-like receptor stimulation. *Clin Transl Immunology* **2018**, *7*, e1036, doi:10.1002/cti2.1036.
24. Kalvegren, H.; Skoglund, C.; Helldahl, C.; Lerm, M.; Grenegard, M.; Bengtsson, T. Toll-like receptor 2 stimulation of platelets is mediated by purinergic P2X1-dependent Ca2+ mobilisation, cyclooxygenase and purinergic P2Y1 and P2Y12 receptor activation. *Thromb Haemost* **2010**, *103*, 398-407, doi:10.1160/th09-07-0442.
25. Anabel, A.S.; Eduardo, P.C.; Pedro Antonio, H.C.; Carlos, S.M.; Juana, N.M.; Honorio, T.A.; Nicolas, V.S.; Sergio Roberto, A.R. Human platelets express Toll-like receptor 3 and respond to poly I:C. *Hum Immunol* **2014**, *75*, 1244-1251, doi:10.1016/j.humimm.2014.09.013.
26. D'Atri, L.P.; Etulain, J.; Rivadeneyra, L.; Lapponi, M.J.; Centurion, M.; Cheng, K.; Yin, H.; Schattner, M. Expression and functionality of Toll-like receptor 3 in the megakaryocytic lineage. *J Thromb Haemost* **2015**, *13*, 839-850, doi:10.1111/jth.12842.
27. Koupenova, M.; Corkrey, H.A.; Vitseva, O.; Manni, G.; Pang, C.J.; Clancy, L.; Yao, C.; Rade, J.; Levy, D.; Wang, J.P., et al. The role of platelets in mediating a response to human influenza infection. *Nat Commun* **2019**, *10*, 1780, doi:10.1038/s41467-019-09607-x.
28. Koupenova, M.; Vitseva, O.; MacKay, C.R.; Beaulieu, L.M.; Benjamin, E.J.; Mick, E.; Kurt-Jones, E.A.; Ravid, K.; Freedman, J.E. Platelet-TLR7 mediates host survival and platelet count during viral infection in the absence of platelet-dependent thrombosis. *Blood* **2014**, *124*, 791-802, doi:10.1182/blood-2013-11-536003.
29. Ward, J.R.; Bingle, L.; Judge, H.M.; Brown, S.B.; Storey, R.F.; Whyte, M.K.; Dower, S.K.; Buttle, D.J.; Sabroe, I. Agonists of toll-like receptor (TLR)2 and TLR4 are unable to modulate platelet activation by adenosine diphosphate and platelet activating factor. *Thromb Haemost* **2005**, *94*, 831-838.
30. Lopes Pires, M.E.; Clarke, S.R.; Marcondes, S.; Gibbins, J.M. Lipopolysaccharide potentiates platelet responses via toll-like receptor 4-stimulated Akt-Erk-PLA2 signalling. *PLoS One* **2017**, *12*, e0186981, doi:10.1371/journal.pone.0186981.
31. Stahl, A.L.; Svensson, M.; Morgelin, M.; Svanborg, C.; Tarr, P.I.; Mooney, J.C.; Watkins, S.L.; Johnson, R.; Karpman, D. Lipopolysaccharide from enterohemorrhagic Escherichia coli binds to platelets through TLR4 and CD62 and is detected on circulating platelets in patients with hemolytic uremic syndrome. *Blood* **2006**, *108*, 167-176, doi:10.1182/blood-2005-08-3219.
32. Kappelmayer, J.; Beke Debreceni, I.; Vida, A.; Antal-Szalmas, P.; Clemetson, K.J.; Nagy, B., Jr. Distinct effects of Re- and S-forms of LPS on modulating platelet activation. *J Thromb Haemost* **2013**, *11*, 775-778, doi:10.1111/jth.12151.
33. Hally, K.E.; La Flamme, A.C.; Harding, S.A.; Larsen, P.D. The effects of aspirin and ticagrelor on Toll-like receptor (TLR)-mediated platelet activation: results of a randomized, cross-over trial. *Platelets* **2019**, *30*, 599-607, doi:10.1080/09537104.2018.1479520.
34. Vallance, T.M.; Ravishankar, D.; Albadawi, D.A.I.; Layfield, H.; Sheard, J.; Vaiyapuri, R.; Dash, P.; Patel, K.; Widera, D.; Vaiyapuri, S. Effect of ultrapure lipopolysaccharides derived from diverse bacterial species on the modulation of platelet activation. *Sci Rep* **2019**, *9*, 18258, doi:10.1038/s41598-019-54617-w.
35. Rex, S.; Beaulieu, L.M.; Perlman, D.H.; Vitseva, O.; Blair, P.S.; McComb, M.E.; Costello, C.E.; Freedman, J.E. Immune versus thrombotic stimulation of platelets differentially regulates signalling pathways, intracellular protein-protein interactions, and alpha-granule release. *Thromb Haemost* **2009**, *102*, 97-110, doi:10.1160/th08-08-0513.
36. Falker, K.; Klarstrom-Engstrom, K.; Bengtsson, T.; Lindahl, T.L.; Grenegard, M. The toll-like receptor 2/1 (TLR2/1) complex initiates human platelet activation via the src/Syk/LAT/PLCgamma2 signalling cascade. *Cell Signal* **2014**, *26*, 279-286, doi:10.1016/j.cellsig.2013.11.011.
37. Klarstrom Engstrom, K.; Khalaf, H.; Kalvegren, H.; Bengtsson, T. The role of Porphyromonas gingivalis gingipains in platelet activation and innate immune modulation. *Mol Oral Microbiol* **2015**, *30*, 62-73, doi:10.1111/omi.12067.
38. Tunjungputri, R.N.; van der Ven, A.J.; Riksen, N.; Rongen, G.; Tacke, S.; van den Berg, T.N.; Fijnheer, R.; Gomes, M.E.; Dinarello, C.A.; van de Veerdonk, F.L., et al. Differential effects of platelets and platelet inhibition by ticagrelor on TLR2- and TLR4-mediated inflammatory responses. *Thromb Haemost* **2015**, *113*, 1035-1045, doi:10.1160/th14-07-0579.
39. Wadowski, P.P.; Weikert, C.; Pultar, J.; Lee, S.; Eichelberger, B.; Koppensteiner, R.; Lang, I.M.; Panzer, S.; Gremmel, T. Ticagrelor Inhibits Toll-Like and Protease-Activated Receptor Mediated Platelet Activation in Acute Coronary Syndromes. *Cardiovascular Drugs and Therapy* **2020**, *34*, 53-63, doi:10.1007/s10557-019-06932-7.

40. Klarstrom Engstrom, K.; Brommesson, C.; Kalvegren, H.; Bengtsson, T. Toll like receptor 2/1 mediated platelet adhesion and activation on bacterial mimetic surfaces is dependent on src/Syk-signaling and purinergic receptor P2X1 and P2Y12 activation. *Biointerphases* **2014**, *9*, 041003, doi:10.1116/1.4901135.
41. Cognasse, F.; Hamzeh, H.; Chavarin, P.; Acquart, S.; Genin, C.; Garraud, O. Evidence of Toll-like receptor molecules on human platelets. *Immunol Cell Biol* **2005**, *83*, 196-198, doi:10.1111/j.1440-1711.2005.01314.x.
42. Shiraki, R.; Inoue, N.; Kawasaki, S.; Takei, A.; Kadotani, M.; Ohnishi, Y.; Ejiri, J.; Kobayashi, S.; Hirata, K.; Kawashima, S., et al. Expression of Toll-like receptors on human platelets. *Thromb Res* **2004**, *113*, 379-385, doi:10.1016/j.thromres.2004.03.023.
43. Koupenova, M.; Mick, E.; Mikhalev, E.; Benjamin, E.J.; Tanriverdi, K.; Freedman, J.E. Sex differences in platelet toll-like receptors and their association with cardiovascular risk factors. *Arterioscler Thromb Vasc Biol* **2015**, *35*, 1030-1037, doi:10.1161/ATVBAHA.114.304954.
44. Aslam, R.; Speck, E.R.; Kim, M.; Crow, A.R.; Bang, K.W.; Nestel, F.P.; Ni, H.; Lazarus, A.H.; Freedman, J.; Semple, J.W. Platelet Toll-like receptor expression modulates lipopolysaccharide-induced thrombocytopenia and tumor necrosis factor-alpha production in vivo. *Blood* **2006**, *107*, 637-641, doi:10.1182/blood-2005-06-2202.
45. Gurses, K.M.; Kocyigit, D.; Yalcin, M.U.; Canpinar, H.; Oto, M.A.; Ozer, N.; Tokgozoglu, L.; Guc, D.; Aytemir, K. Enhanced Platelet Toll-like Receptor 2 and 4 Expression in Acute Coronary Syndrome and Stable Angina Pectoris. *Am J Cardiol* **2015**, *116*, 1666-1671, doi:10.1016/j.amjcard.2015.08.048.
46. Marín Oyarzún, C.P.; Glembotsky, A.C.; Goette, N.P.; Lev, P.R.; De Luca, G.; Baroni Pietto, M.C.; Moiraghi, B.; Castro Ríos, M.A.; Vicente, A.; Marta, R.F., et al. Platelet Toll-Like Receptors Mediate Thromboinflammatory Responses in Patients With Essential Thrombocythemia. *Frontiers in Immunology* **2020**, *11*, doi:10.3389/fimmu.2020.00705.
47. Heger, L.A.; Hortmann, M.; Albrecht, M.; Colberg, C.; Peter, K.; Witsch, T.; Stallmann, D.; Zirlik, A.; Bode, C.; Duerschmied, D., et al. Inflammation in acute coronary syndrome: Expression of TLR2 mRNA is increased in platelets of patients with ACS. *PLoS One* **2019**, *14*, e0224181, doi:10.1371/journal.pone.0224181.
48. Andonegui, G.; Kerfoot, S.M.; McNagny, K.; Ebbert, K.V.; Patel, K.D.; Kubes, P. Platelets express functional Toll-like receptor-4. *Blood* **2005**, *106*, 2417-2423, doi:10.1182/blood-2005-03-0916.
49. Wang, Y.Q.; Wang, B.; Liang, Y.; Cao, S.H.; Liu, L.; Xu, X.N. Role of platelet TLR4 expression in pathogensis of septic thrombocytopenia. *World J Emerg Med* **2011**, *2*, 13-17, doi:10.5847/wjem.j.1920-8642.2011.01.002.
50. Larkin, C.M.; Hante, N.K.; Breen, E.P.; Tomaszewski, K.A.; Eisele, S.; Radomski, M.W.; Ryan, T.A.; Santos-Martinez, M.J. Role of matrix metalloproteinases 2 and 9, toll-like receptor 4 and platelet-leukocyte aggregate formation in sepsis-associated thrombocytopenia. *PLoS One* **2018**, *13*, e0196478, doi:10.1371/journal.pone.0196478.
51. Schmid, W.; Novacek, G.; Vogelsang, H.; Papay, P.; Primas, C.; Eser, A.; Panzer, S. Platelets Toll-like receptor-4 in Crohns disease. *Eur J Clin Invest* **2017**, *47*, 109-116, doi:10.1111/eci.12686.
52. Assinger, A.; Laky, M.; Badrnya, S.; Esfandeyari, A.; Volf, I. Periodontopathogens induce expression of CD40L on human platelets via TLR2 and TLR4. *Thromb. Res.* **2012**, *130*, e73-e78, doi:10.1016/j.thromres.2012.04.017.
53. Leroy, J.; Bortolus, C.; Lecointe, K.; Parny, M.; Charlet, R.; Sendid, B.; Jawhara, S. Fungal Chitin Reduces Platelet Activation Mediated via TLR8 Stimulation. *Frontiers in Cellular and Infection Microbiology* **2019**, *9*, doi:10.3389/fcimb.2019.00383.
54. Thon, J.N.; Peters, C.G.; Machlus, K.R.; Aslam, R.; Rowley, J.; Macleod, H.; Devine, M.T.; Fuchs, T.A.; Weyrich, A.S.; Semple, J.W., et al. T granules in human platelets function in TLR9 organization and signaling. *J Cell Biol* **2012**, *198*, 561-574, doi:10.1083/jcb.201111136.
55. Hally, K.E.; La Flamme, A.C.; Larsen, P.D.; Harding, S.A. Toll-like receptor 9 expression and activation in acute coronary syndrome patients on dual anti-platelet therapy. *Thromb Res* **2016**, *148*, 89-95, doi:10.1016/j.thromres.2016.10.026.
56. Berthet, J.; Damien, P.; Hamzeh-Cognasse, H.; Arthaud, C.-A.; Eyraud, M.-A.; Zéni, F.; Pozzetto, B.; McNicol, A.; Garraud, O.; Cognasse, F. Human platelets can discriminate between various bacterial LPS isoforms via TLR4 signaling and differential cytokine secretion. *Clinical Immunology* **2012**, *145*, 189-200, doi:10.1016/j.clim.2012.09.004.
57. Jerez-Dolz, D.; Torramade-Moix, S.; Palomo, M.; Moreno-Castano, A.; Lopez-Vilchez, I.; Hernandez, R.; Badimon, J.J.; Zafar, M.U.; Diaz-Ricart, M.; Escolar, G. Internalization of microparticles by platelets is partially mediated by toll-like receptor 4 and enhances platelet thrombogenicity. *Atherosclerosis* **2020**, *294*, 17-24, doi:10.1016/j.atherosclerosis.2019.12.017.
